# International Exercise Recommendations in Older Adults (ICFSR): Expert Consensus Guidelines

**DOI:** 10.1007/s12603-021-1665-8

**Published:** 2021-07-30

**Authors:** Mikel Izquierdo, R.A. Merchant, J.E. Morley, S.D. Anker, I. Aprahamian, H. Arai, M. Aubertin-Leheudre, R. Bernabei, E.L. Cadore, M. Cesari, L.-K. Chen, P. de Souto Barreto, G. Duque, L. Ferrucci, R.A. Fielding, A. García-Hermoso, L.M. Gutiérrez-Robledo, S.D.R. Harridge, B. Kirk, S. Kritchevsky, F. Landi, N. Lazarus, F.C. Martin, E. Marzetti, M. Pahor, R. Ramírez-Vélez, L. Rodriguez-Mañas, Y. Rolland, J.G. Ruiz, O. Theou, D.T. Villareal, D.L. Waters, C. Won Won, J. Woo, B. Vellas, M. Fiatarone Singh

**Affiliations:** 1 Navarrabiomed, Complejo Hospitalario de Navarra (CHN), Universidad Pública de Navarra (UPNA), IdiSNA, Pamplona, Spain; 2 CIBER of Frailty and Healthy Ageing (CIBERFES) Instituto de Salud Carlos III, Madrid, Spain; 3 Division of Geriatric Medicine, Department of Medicine, National University Hospital, Singapore, Singapore; 4 Department of Medicine, Yong Loo Lin School of Medicine, National University Singapore, Singapore, Singapore; 5 Saint Louis University School of Medicine, St. Louis, MO, USA; 6 Department of Cardiology (CVK); and Berlin Institute of Health Center for Regenerative Therapies (BCRT); German Centre for Cardiovascular Research (DZHK) partner site Berlin, Charité Universitätsmedizin Berlin, Berlin, Germany; 7 Department of Internal Medicine, Division of Geriatrics, Faculty of Medicine of Jundiaí, Jundiaí, Brazil; 8 National Center for Geriatrics and Gerontology, Obu, Japan; 9 Département des Sciences de l'Activité Physique, Groupe de Recherche en Activité Physique Adapté, Université du Québec à Montréal (UQAM), Montréal, Qc, Canada; 10 Centre de Recherche de l'Institut Universitaire de Gériatrie de Montréal (CRIUGM), Montréal, Qc, Canada; 11 Department of Geriatrics, Neurosciences and Orthopedics, Università Cattolica del Sacro Cuore and Fondazione Policlinico Universitario «Agostino Gemelli» IRCCS, Rome, Italy; 12 Exercise Research Laboratory, School of Physical Education, Physiotherapy and Dance, Universidade Federal do Rio Grande do Sul, Porto Alegre, Brazil; 13 IRCCS Istituti Clinici Scientifici Maugeri, Università degli Studi di Milano, Milan, Italy; 14 Aging and Health Research Center, National Yang Ming Chiao Tung University, Taipei, Taiwan; 15 Institute of Neuroscience, National Yang Ming Chiao Tung University, Taipei, Taiwan; 16 Center for Geriatrics and Gerontology, Taipei Veterans General Hospital, Taipei, Taiwan; 17 Taipei Municipal Gan-Dau Hospital, Taipei, Taiwan; 18 Gérontopôle de Toulouse, Institut du Vieillissement, Centre Hospitalo-Universitaire de Toulouse, 37 allées Jules Guesdes, 31000, Toulouse, France; 19 CERPOP, Inserm 1295, Université de Toulouse, UPS, 31000, Toulouse, France; 20 Australian Institute for Musculoskeletal Science (AIMSS), The University of Melbourne and Western Health, St Albans, Melbourne, Victoria, Australia; 21 Department of Medicine-Western Health, Melbourne Medical School, University of Melbourne, St Albans, Melbourne, VIC, Australia; 22 National Intitute on Aging, Baltimore, MD, USA; 23 Nutrition, Exercise Physiology, and Sarcopenia Laboratory, Jean Mayer USDA Human Nutrition Research Center on Aging, Tufts University, 02111, Boston, MA, USA; 24 Instituto Nacional de Geriatría, Mexico City, Mexico; 25 Centre for Human and Applied Physiological Sciences, King's College London, London, UK; 26 Wake Forest School of Medicine, Sticht Center for Healthy Aging and Alzheimer's Prevention, Winston-Salem, NC, USA; 27 School of Population Health & Environmental Sciences, Faculty of Life Sciences and Medicine, King's College London, London, UK; 28 Department of Aging and Geriatric Research, Institute on Aging, University of Florida College of Medicine, Gainesville, FL, USA; 29 Geriatric Service, University Hospital of Getafe, Getafe, Spain; 30 Miami VAHS GRECC and University of Miami Miller School of Medicine, Miami, USA; 31 Physiotherapy and Geriatric Medicine, Dalhousie University, Halifax, Nova Scotia, Canada; 32 Baylor College of Medicine, Houston, Texas, USA; 33 Department of Medicine, School of Physiotherapy, University of Otago, Dunedin, New Zealand; 34 Elderly Frailty Research Center, Department of Family Medicine, College of Medicine, Kyung Hee University, Seoul, Republic of Korea; 35 Department of Medicine and Therapeutics, and Jockey Club Institute of Ageing, The Chinese University of Hong Kong, Hong Kong, China; 36 Faculty of Medicine and Health, School of Health Sciences and Sydney Medical School, University of Sydney, Sydney, New South Wales, Australia; 37 Hinda and Arthur Marcus Institute for Aging Research, Hebrew SeniorLife, Roslindale, MA, USA; 38 Department of Health Sciences, Public University of Navarra, Av. De Barañain s/n, 31008, Pamplona, Navarra, Spain

**Keywords:** Sarcopenia, frail, falls, exercise, functional capacity, multicomponent training, diseases

## Abstract

The human ageing process is universal, ubiquitous and inevitable. Every physiological function is being continuously diminished. There is a range between two distinct phenotypes of ageing, shaped by patterns of living - experiences and behaviours, and in particular by the presence or absence of physical activity (PA) and structured exercise (i.e., a sedentary lifestyle). Ageing and a sedentary lifestyle are associated with declines in muscle function and cardiorespiratory fitness, resulting in an impaired capacity to perform daily activities and maintain independent functioning. However, in the presence of adequate exercise/PA these changes in muscular and aerobic capacity with age are substantially attenuated. Additionally, both structured exercise and overall PA play important roles as preventive strategies for many chronic diseases, including cardiovascular disease, stroke, diabetes, osteoporosis, and obesity; improvement of mobility, mental health, and quality of life; and reduction in mortality, among other benefits. Notably, exercise intervention programmes improve the hallmarks of frailty (low body mass, strength, mobility, PA level, energy) and cognition, thus optimising functional capacity during ageing. In these pathological conditions exercise is used as a therapeutic agent and follows the precepts of identifying the cause of a disease and then using an agent in an evidence-based dose to eliminate or moderate the disease. Prescription of PA/structured exercise should therefore be based on the intended outcome (e.g., primary prevention, improvement in fitness or functional status or disease treatment), and individualised, adjusted and controlled like any other medical treatment. In addition, in line with other therapeutic agents, exercise shows a dose-response effect and can be individualised using different modalities, volumes and/or intensities as appropriate to the health state or medical condition. Importantly, exercise therapy is often directed at several physiological systems simultaneously, rather than targeted to a single outcome as is generally the case with pharmacological approaches to disease management. There are diseases for which exercise is an alternative to pharmacological treatment (such as depression), thus contributing to the goal of deprescribing of potentially inappropriate medications (PIMS). There are other conditions where no effective drug therapy is currently available (such as sarcopenia or dementia), where it may serve a primary role in prevention and treatment. Therefore, this consensus statement provides an evidence-based rationale for using exercise and PA for health promotion and disease prevention and treatment in older adults. Exercise prescription is discussed in terms of the specific modalities and doses that have been studied in randomised controlled trials for their effectiveness in attenuating physiological changes of ageing, disease prevention, and/or improvement of older adults with chronic disease and disability. Recommendations are proposed to bridge gaps in the current literature and to optimise the use of exercise/PA both as a preventative medicine and as a therapeutic agent.

## Physical activity and exercise for health promotion, disease prevention and treatment in older adults

**T**he world population is ageing, and adults ≥ 65 years old are projected to double in number to ∼ 1.5 billion in 2050. Due to a concurrent increase in life expectancy, people ≥ 80 years of age are projected to triple in number between 2019 and 2050 to 426 million (1). Population ageing impacts many sectors, including healthcare, quality of life (social), retirement, caregiving, and most importantly, is associated with an age-related burden of non-communicable chronic diseases and disability. The human ageing process is universal, ubiquitous, inevitable and decremental. Every physiological function is being continuously diminished. At around 20–30 years of age humans have acquired all the physiological development that they will attain. From that stage the ageing process commences, although the rate of change is heterogeneous. This process will continue up to six or seven decades ending in death.

There is a range between two distinct phenotypes of ageing, shaped by patterns of living — experiences and behaviours, and in particular by the presence or absence of ‘healthful’ levels of physical activity (PA) and exercise (2, 3). In this consensus, the terms ‘physical activity’ and ‘exercise’ should be interpreted as follows: PA is any bodily movement produced by skeletal muscles that significantly increases energy expenditure (4). The intensity and duration of PA can vary substantially. Exercise is a subcategory of PA that is planned, structured, and repetitive, in which bodily movements are performed with or without the explicit intent of improving or maintaining of one or more components of physical fitness (i.e., aerobic capacity, muscle strength power and endurance, balance, coordination, and flexibility) (5).

### Distinctive phenotypes of Ageing

In the presence of PA lifestyle-related diseases such as cardiometabolic disease, obesity, and cerebrovascular disease, for example, are prevented or ameliorated. Exercise may be used here as a preventative measure in conjunction with other lifestyle factors such as diet. At one end of the PA/ exercise continuum lie lifelong exercisers and competitive master athletes. Despite the decline in their competitive performances (as evidenced by the decline in world records) the absolute levels of physical performance in such individuals are remarkable when compared to age-matched sedentary peers (6). Such individuals show the power of exercise (as well as favourable genetic profile potentially) in the maintenance of physiological function, and arguably represent the peak potential for human health and functional ability into old age. Towards the other end of the spectrum and in the absence of adequate PA/exercise, the ageing process is associated with the premature and excessive appearance of disease and dysfunction These diseases contributing substantially to all-cause mortality and include cardiovascular diseases, type 2 diabetes, obesity, diminished muscular function, mental health problems, and increased end of life morbidity. Physical activity operates as preventive medicine in the minimal disease phenotype, while in the lifestyle-related disease phenotype, PA tends to be applied far downstream from the initiation of pathological processes and therefore operates as a true therapeutic agent, where it follows the precepts of identifying the cause of a disease and then using an agent to eliminate or moderate the disease (2, 3).

**Figure 1:**
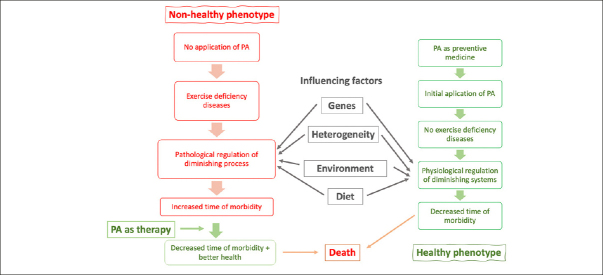
The left-hand side shows the application of PA as a therapeutic agent to pathologies that have developed because of a lack of exercise. This side reflects pathological, not physiological changes. The effects of these pathologies can be ameliorated by PA. The right-hand side show the application of PA as preventative medicine and the maintenance of effective but diminishing physiological function. This side is investigated by the science of physiology and ageing. Both sides are in constant decremental change because of the inherent ageing process. The centre shows major effectors of both pathological and physiological processes

However, a choice is available for many. This choice will have a great influence on ageing trajectories. There are many factors that make up a lifestyle but in the context of this article it is the effect of PA/exercise that is of particular interest, as it optimises physiology when engaged in with adequate doses and modalities. Although socioeconomic conditions do not always allow for this, many people have the opportunity to adopt a lifestyle that will either give protection from these diseases or an alternate lifestyle that will make them more susceptible to lifestyle diseases (Figure 1).

Figure 1. The left-hand side shows the application of PA as a therapeutic agent to pathologies that have developed because of a lack of exercise. This side reflects pathological, not physiological changes. The effects of these pathologies can be ameliorated by PA. The right-hand side show the application of PA as preventative medicine and the maintenance of effective but diminishing physiological function. This side is investigated by the science of physiology and ageing. Both sides are in constant decremental change because of the inherent ageing process. The centre shows major effectors of both pathological and physiological processes.

**Figure 2:**
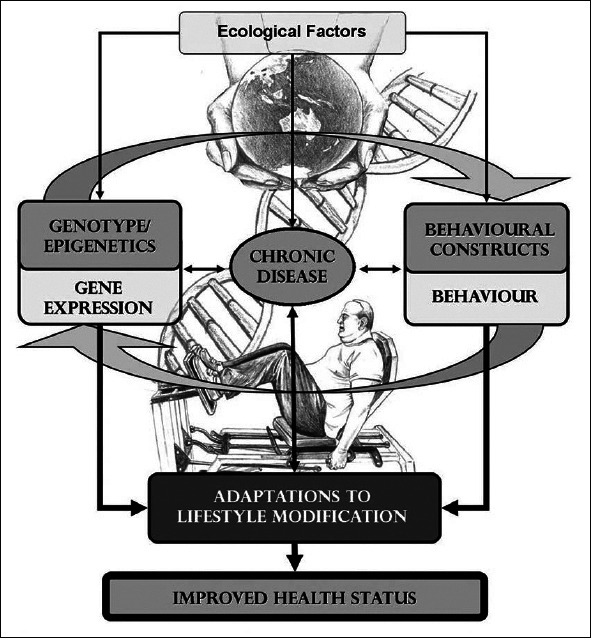
The interaction between an individual's intrinsic characteristics, behaviours and environmental/ecological influences are crucial to achieving the optimum trajectory which can be modified to maintain a person's functional ability and intrinsic capacity throughout the life course

While ageing is the major risk factor for most chronic diseases, the relationship is bidirectional, as chronic diseases, the so-called geriatric syndromes, and/or adverse consequences of their treatment may accelerate biological ageing. Among the most important of these conditions are frailty, sarcopenia and dementia, which are all precursors of disability and accelerated ageing (7). In 2015, the World Report on Ageing and Health by World Health Organisation (WHO) defined healthy ageing as the process of developing and maintaining functional ability (8). The interaction between an individual's intrinsic characteristics, behaviours and environmental/ecological influences are crucial to achieving the optimum trajectory, which can be modified to maintain a person's functional ability and intrinsic capacity throughout the life course, but especially in old age when the resilience strategies aimed at avoiding damage accumulation become less efficient (Figure 2).

The main factors that influence health and longevity include genetics, environment, and behaviour, all of which can modify the expression of the other. Epigenetic modification of the genome via lifestyle factors or exposure to stress as early as in utero are among the increasingly recognised examples of the inextricable inter-dependence of nature and nurture, and contribute to the manifestations of ageing, and in chronic diseases or multimorbidity [9, 10]. Among modifiable factors associated with beneficial effects across the life span, insufficient physical activity (PA) and sedentary lifestyle are among the most significant public health problems which, according to the WHO, should be corrected to promote healthy ageing [11]. Although many questions remain unanswered about the mechanisms of the beneficial health effects of PA and the optimal modalities and intensity, a synthesis of the literature indicates multiple positive effects of participation in PA on the ageing process and mortality, with dose-response curves showing that health benefits are linked to both the intensity and volume of exercise [12].

Research evidence shows that PA (including structured exercise participation) influences key drivers of healthy ageing even in the oldest-old, including chronic mitochondrial dysfunction, inflammation, myokine release, defective autophagy, oxidative damage and reduced insulin-like growth factor signalling (13, 14). Exercise and PA improve physical function and quality of life which overall reduce the burden of non-communicable diseases and premature overall mortality, including specific mortality causes such as cardiovascular disease, cancer, and chronic lower respiratory tract diseases (15, 16, 17, 18). There is robust scientific evidence for the benefits of PA and exercise in the prevention and treatment of many chronic diseases such as sarcopenia, diabetes, cardiovascular disease, hypertension, cancer, osteoporosis, osteoarthritis, depression, dementia or Parkinson's disease, among many others (18).

The effects of PA on ageing and chronic diseases are closely related to the attenuation of adverse age-related changes in body composition. Ageing is associated with significant bone and muscle mass losses, and increases in adipose tissue, along with critical shifts in the distribution of adipose tissue to more central and visceral depots rather than appendicular and subcutaneous depots. Intramuscular, peri-hepatic and intrahepatic adipose tissue as well as pericardial adipose tissue accumulation with ageing (but also sedentary lifestyles) are examples of this “unhealthy” adiposity which is linked to biological background of metabolic and functional impairments (muscle mass and strength loss), type 2 diabetes, cardiovascular disease, and many other conditions (14, 19).

In addition to changes in body composition, declines in exercise capacity with ageing, inactivity and sedentary lifestyles have significant health-related consequences. Declines in muscle function and cardiorespiratory fitness with ageing result in impaired ability to perform daily activities and maintain independence (14, 19). They are also associated with cognitive decline, especially in reasoning, processing speed, attention, executive function, and memory, due to structural changes in the frontal and medial temporal lobes including the hippocampus and amygdala. In addition to the impact of exercise on improving muscle strength, muscle quality, muscle mass, bone density, and mobility in older adults, exercise also has beneficial effects on cognitive function. These cognitive exercise benefits appear to be mediated in part through its influence on brain-derived neurotrophic factor (BDNF), alteration in cerebral blood flow and functional connectivity, with associated structural changes involving white and grey matter integrity, posterior cingulate cortex thickness and the hippocampal size (20, 21, 22, 23, 24).

As is the case for all ages, the prescription of exercise for health-related outcomes must consider dose-response relationships with volume and intensity, but also modality-specific adaptations that are requisite for specific outcomes to be achieved. For example, resistance, aerobic, balance, and mobility training can address specific age-related deficits. Multicomponent exercise intervention programmes that include a cognitive task effectively improve the hallmarks of frailty (low body mass, strength, endurance mobility, physical activity level, energy) and cognition, thus optimising functional capacity during ageing (25, 26). Muscle power output (the product of force of contraction and speed of movement) and physical function during tasks of everyday living are closely associated. The very dramatic loss of muscle power specifically with ageing (due to fast-twitch /type II fibre atrophy and changes in neural recruitment), provides a rationale for explosive resistance training (known as power training and characterized by fast and forceful muscle contractions) in the exercise prescription whenever possible to optimise functional outcomes in both fit and frail older adults (27, 28). Sarcopenia (the loss of muscle mass, strength, and function with ageing) requires high-intensity resistance training for optimal adaptation, demonstrating the clear need to consider both modality and intensity when addressing this pervasive disease (29).

There is inter-individual heterogeneity in physiological responses and adaptions to PA and/or exercise, and there are many ongoing discussions regarding the classification of “responder”, “non-responder”, or “adverse responder”. Exercise is medicine, and the prescription of PA/structured exercise should therefore be based on the intended outcome (e.g., primary prevention, improvement in fitness or functional status or disease treatment), and should be individualised, adjusted, and controlled like any other medical treatment. The dosage recommendations should consider the external (exercise variable), and internal (acute response to exercise) loads which are influenced by personal, genetic, functional, psychosocial factors and the external environment [30] (see Figure 2). Unfortunately, we do not have enough information for precision prescription, and this remains an important area of research.

In summary, this consensus statement attempts to provide a rationale for using exercise and/or PA for health promotion, disease prevention and treatment in older adults. This paper includes evidence from randomised controlled trials demonstrating the favourable effects of specific PA/Exercise modalities on age-related physiological changes, disease prevention, and treatment of older adults with chronic disease and disability. We offer recommendations to address knowledge gaps and clinical implementation needs in this field.

## Physical activity and exercise: Global recommendations for health

Strategies aimed at increasing population-level PA and optimising its adherence have focused on promoting exercise via “lifestyle integration” by incorporating it into activities of daily life. For example, taking the stairs instead of the elevator, standing on one leg while doing the dishes, or slowly standing and sitting without using of the arms represent ways of incorporating aerobic, balance, and strengthening exercises, respectively, into everyday activities. Current investigations explore whether such prescriptive techniques can achieve better exercise adherence compared to standard approaches for promoting behavioural change and targeting clinical outcomes such as falls in older adults (31, 32).

The WHO's ‘Global Recommendations on Physical Activity for Health’ state that adults 65 and older should engage in 150 minutes of moderate- or 75 minutes of vigorous-intensity aerobic activity and two or more days of muscle-strengthening activity (i.e., strength/resistance training) per week (33). The US Department of Health and Human Services (HHS) suggests that multicomponent exercises training that includes balance training as well as muscle-strengthening (at least 2 days a week) and aerobic activities of at least moderate-intensity performed 3 or more times per week for a duration of 30 to 45 minutes per session over at least 3 to 5 months appears most effective to increase functional ability in older adults with frailty (34). However, current PA guidelines are rarely met, particularly in older adults. For example, from 2015 to 2019 the proportion of U.S adults meeting both aerobic and resistance exercise guidelines (defined as moderate-vigorous aerobic activity ≥ 150 minutes/week and resistance training ≥ 2 sessions/week) is not high. An analysis of the Behavioural Risk Factor Surveillance Survey of ∼ 350,000 adults aged 18–80 years in the USA showed only a slight increase in the proportion of people meeting both guidelines from 2015 – 2019 (from 17 to 23%) (35, 36). Insufficient PA combined with a sedentary lifestyle which often accompany ageing are antecedents for sarcopenia, frailty, obesity and chronic diseases (3, 37, 38, 39, 40). Estimates from 2017 indicate that not meeting PA recommendations is responsible for approximately 1.3 million deaths (17 deaths per 100,000 inhabitants) in individuals aged 25 years and over globally each year (41).

The role of exercise in the prevention of disease and the management of several age-related diseases and conditions is increasingly evident, including syndromes for which the benefit of pharmacological treatment is controversial. There is strong evidence for PA and exercise as both a preventive and therapeutic strategy for cardiovascular disease, diabetes, and obesity; improvement of muscular function, mental health, and quality of life; and reduction in mortality (42, 43). Similarly, the combination of balance and resistance training is the most effective intervention for reducing falls, for which pharmacological therapy is unavailable (3, 44, 45, 46) and resistance training is the core treatment for sarcopenia (14, 47), whereas drugs have not shown clear benefits (48, 49). There seems little logic in using a pharmaceutical chemical directed to a specific muscle target in a diminished muscle when the pathology lies not only in the muscle but also in all the pathways from the central nervous system to the periphery. Similarly, there is no effective pharmacological treatment to slow down ageing and associated frailty, disability, cognitive decline. Importantly, even when we have medications at our disposal for the treatment of common psychological problems such as depression, insomnia, and anxiety (antidepressants, insomniacs, and anxiolytics, respectively), these potentially inappropriate medications (PIMs) may be associated with adverse drug events (ADEs) such as falls, hip fractures, delirium, and worsening of cognitive impairment, which is far less desirable than the use of evidence-based exercise prescriptions to treat these conditions [50]. Thus, the actual utility of exercise as medicine is four-fold: 1) to exploit its potential to prevent diseases for which we have available treatments; 2) to serve as an adjunct to effective medical/ surgical interventions where they do exist; 3) to substitute for hazardous treatments for which exercise represents a better and safer alternative; and 4) to become mainstream in the management of conditions for which there is no other effective treatment. The latter category represents the most pervasive and morbid conditions of older adults globally: sarcopenia, frailty, disability, and dementia.

Despite its multiple benefits, exercise is not fully integrated into geriatric medicine practice. It is still absent from the core training of most geriatricians and other healthcare professionals (13, 32, 51). In addition, few studies have explored the potential role of tailored PA guidelines to maximise exercise-related effects on function, ability to perform activities of daily living, or on other domains of intrinsic capacity such as cognitive, psychological, or sensory deficits (e.g., vision or hearing), and locomotion or vitality in older adults, which is likely to be related to the paucity of research in the area (32). Tailored interventions for increasing population-level PA should also consider behavioural and social aspects to ensure adherence and increase motivation for PA (e.g., to emphasise the wide range of benefits associated with a physically active lifestyle) and enhance self-efficacy (32, 52, 53). Effectively targeting not only behavioural causes of ill health and inequality (e.g., tobacco use, alcohol consumption, unhealthy diet, and physical inactivity) but also boosting social and environmental support for exercise to improve older adults' PA levels is vitally important. For example, the implementation of strategies to improve access to PA facilities, and modifying public and private spaces (e.g., work sites) to promote PA and to reduce sedentary behaviour is needed (32, 54).

### Preserving exercise capacity with age via habitual engagement in physical activity/exercise

Many studies suggest that habitual engagement in PA/ exercise can markedly attenuate most decrements in exercise capacity that are typically attributed to ageing. A notable exception is the decline of maximal heart rate due to decreased sensitivity to β-adrenergic stimulation in the ageing heart (55). Although aged individuals achieve lower peak exercise workloads, cardiovascular and musculoskeletal adaptations to chronic aerobic exercise enable trained individuals to sustain higher submaximal workloads with both lower cardiorespiratory responses (heart rate, blood pressure, and dyspnoea) and overall musculoskeletal fatigue. Although there is with some overlap, exercise adaptations are specific to the selected modality chosen. Benefits in aerobic capacity are best achieved with moderate- to- vigorous-intensity aerobic exercise (MICT), with the greatest effects seen with high-intensity interval training (HIIT, 85–95% peak heart rate for 1–4 minutes intervals) (56). Higher load resistance or strength training represents the optimal prescription to treat sarcopenia and may also improve balance (57). Less well-known is that there is evidence that high-intensity progressive resistance training in older adults also improves aerobic capacity to a similar extent as moderate-intensity aerobic training (58, 59). Thus, when initiating exercise in stages it may be easier behaviourally to provide a single exercise prescription consisting of a resistance exercise, which would target two major age-related changes in exercise capacity, before adding other modalities. By contrast, aerobic exercise alone does not improve strength nor balance and thus is insufficient as a single modality for older adults. Systematic reviews clearly indicate that falls-prevention programmes that include walking as a single modality are inferior to those combining strength and balance exercise and such interventions have been linked to higher falls and osteoporotic fracture rates in individuals at risk (45).

Similar to aerobic and resistance training, there is evidence that balance and flexibility training induce specific adaptations in these health-related physical fitness components (60). Improvement of balance is linked to a reduction in falls and falls-related injuries and improvements in functional mobility (45, 61, 62). Although stretching is generally included in most position statements (5, 63), there is limited evidence that improvements in flexibility by themselves are associated with any important clinical outcomes. Flexibility exercise is best conceptualised as a component of cool-down routines after the actual exercise session has been completed. Stretching prior to exercise has not been shown to reduce musculoskeletal injuries as once thought and may result in reduced post-stretching muscle performance. The best warm-up for cardiovascular and musculoskeletal systems is simply to do what is about to be done but at a lower intensity. This may mean, for example, walking at a slow pace or performing a set of weightlifting repetitions with lighter loads (51).

Therefore, it is vital to promote healthy and dignified ageing by assisting healthcare systems more efficiently to implement evidence-based exercise programmes for older adults across levels of frailty in community and institutional settings. In the sections that follow, the paper will focus on changes in functional capacity, physical fitness, body composition, quality of life, and disease burden, rather than on overall changes in the longevity process itself. It is in these domains that the centrality of PA patterns to achieve optimal ageing is perhaps most relevant to the concerns of the healthcare professional and the older individual.

## Evidence for specific modalities of exercise in older adults

There are sufficient data from both epidemiological studies and experimental trials to support the training of all physicians, including geriatricians (51), in the basics of exercise prescription for health-related quality of life (Tables 1). As mentioned earlier, there is strong evidence that exercise training is effective in the management of most of the major non-communicable chronic diseases and associated co-morbidities, such as cognitive impairment frailty, sarcopenia, falls, and mobility impairment (18, 39).

**Table 1:** Exercise recommendations for optimal ageing and maintenance of functional capacities in older adults

	**Resistance Training**	**Aerobic Exercise Training**	**Balance Training**
Frequency (days per week)	2 – 3	3 – 7	1 – 7
Volume	1–3 sets of 8–12 repetitions, 8–10 major muscle groups	20 – 60 minutes / session	1 – 2 sets of 4 – 10 different exercises emphasizing static and dynamic postures
Intensity	Start at 30–40% of 1RM and progress to heavier loads of 70–80% 1 RM (15–18 on Borg Scalea) 1–3 min rest between sets Power training at 40 – 60% of 1RM	12–14 on Borg Scale^a^ (55–70% heart rate reserve or maximum exercise capacity)	Progressive difficulty as tolerated^b^ Narrowing the base of support Perturbation of ground support Decrease in proprioceptive sensation Diminished or misleading visual inputs Movement of the centre of mass of the body away from the vertical or stationary position Dual tasking: adding a cognitive distractor or secondary physical task while practising a balance task
Specific Physiological adaptations	• Strength • Power • Hypertrophy • Endurance • Maximal aerobic capacity	• Maximal aerobic capacity • Sub-maximal endurance • Cardiac contractility/stroke volume • Peripheral oxygen extraction • Arterial stiffness • Heart rate variability	• Dynamic stability
Exercise examples	• Multiple and single joint exercises (free weights and machine) with slow to moderate lifting velocity • Bench press and squat • Knee extensions and knee curls • Exercise selection can be varied through alterations in body posture, grip, hand and foot stance, unilateral vs bilateral exercises • Once body weight no longer serves as a sufficient source of overload, additional resistance can be provided by machines or free weights as needed to ensure progression.	• Dancing • Cycling • Hiking • Jogging / long distance running • Swimming • Walking with change in pace and direction • Treadmill walking • Stair climbing • Step-ups • Seated stepping • Recumbant cycling May start with 5–10 mins and progress to 15–30 mins. The intensity is proportional to heart rate and/or perceived exertional scales if on B blockers or has pacemaker and can be increased from moderate to vigorous depending on fitness.	• Tai Chi • Standing yoga or ballet movements • Tandem walking • Standing on one leg, stepping over objects, climbing slowly up and down steps • Turning • Standing on heels and toes, walking on a compliant surface such as foam mattresses • Maintaining balance on a moving vehicle, such as a bus or train • Dual-tasking: adding cognitive distractor while maintaining balance Many conditions in older adults require balance training before aerobic exercise/ gait retraining


a. Original Borg Scale of Perceived Exertion from 6 (easy) to 20 (maximal); b. Intensity is increased by decreasing the base of support [e.g., progressing from standing on two feet while holding on to the back of a chair to standing on one foot with no hand support); by decreasing other sensory input (e.g., closing eyes or standing on a foam pillow); by perturbing the centre of mass (e.g., holding a heavy object out to one side while maintaining balance, standing on one leg while lifting the other leg out behind the body, or leaning forward as far as possible without falling or moving feet); or by dual-tasking (adding a secondary cognitive [e.g., naming animals] or physical (e.g., juggling) task while tandem walking].

Screening for sedentary behaviour and insufficient PA should occur during most encounters with healthcare professionals, given their roles as potent risk factors for all-cause and cardiovascular mortality, obesity, sarcopenia, hypertension, insulin resistance, cardiovascular disease, diabetes, stroke, colon cancer, depression, dementia, osteoarthritis, osteoporosis, recurrent falls, frailty, and disability, among other conditions. An appropriate exercise prescription should be included in all healthcare recommendations (13, 32).

Exercise advice should be individualised, referenced to the intended outcomes, and personalized regarding the modality, frequency, duration, and intensity including practical implementation solutions and behavioural support systems to monitor outcome and provide feedback. Although progress has been made in integrating exercise counselling during healthcare encounters with community-dwelling older adults, the advice is often limited to those individuals without significant physical or mental impairments. Considering the accumulated evidence on the benefits of exercise in older adults across levels of frailty, it is no longer justifiable to omit the exercise prescription from clinical encounters. One of the main challenges for the future is to integrate exercise programmes as a mandatory component of the care of older patients with frailty who are in hospital, outpatient clinic, or institutional care settings (40).

Current position statements and consensus guidelines for PA in older adults generally recommend a multi-modal exercise prescription that includes aerobic, strengthening, balance, and flexibility training, via a combination of structured and incidental (lifestyle-integrated) activities (5, 47, 51, 64). However, to achieve optimal levels of adherence and minimise attrition, it would be wise to start with a single exercise modality to allow the sedentary older adult to gradually adapt to the new exercise routine before adding other components (65). It has also been recognized that amounts of exercise below general guidelines can also lead to some health benefits (66). Given the well-known curvilinear relationship between mortality risk and exercise volume (12, 67), even a small amount of exercise is better than no exercise. The exercise prescription should be individualised considering risk factors, medical history, musculoskeletal limitations, functional ability, tolerance, and personal preferences. If significant deficits in muscle strength or balance are identified, these should ideally be addressed before the initiation of aerobic training. Prescribing aerobic training alone will not provide optimal benefits for the musculoskeletal system. Rather, the training programme should be multicomponent involving strength, gait and balance exercises to reduce health outcomes associated with frailty and sarcopenia such as falls and fractures.

A few generalisations can be made, as follows:

• Sequencing: Sequence exercise in people living with severe frailty the same way as the physical requirements underpinning mobility: standing up requires strength and power, staying upright involves balance, and walking any distance requires endurance. This sequence then follows a logic progression. Attempting to ambulate those who cannot lift their body weight out of a chair or maintain standing balance is likely to fail and increase risk of falls (51).
• Paying attention to the physiological determinants of transfer ability and ambulation and targeting these specifically with the appropriate exercise prescription when reversible deficits are uncovered is most likely to succeed. For example, triceps /elbow extensor strength is critical to transfer ability, and improving it has been linked to reduced nursing home admission after hip fracture (68).
• In some cases, a chronic health condition may benefit equally from resistance or aerobic training (e.g., as in the treatment of depression). Still, the decision made is based on the ability to tolerate one form of exercise over another, or the presence of a second disease which requires a specific prescription. Severe osteoarthritis of the knee, recurrent falls, and a low threshold for ischaemia may make resistance training safer and more tolerable than aerobic training as an antidepressant treatment, for example.
• Prioritisation requires careful consideration of the risks and benefits of each mode of activity, as well as the current health status and physical fitness level. If one modality of exercise addresses multiple conditions, it is preferable to one that is more limited. For example, in patients with osteoporosis and depression, resistance training is a more logical choice than aerobic exercise, which evidence suggests can only address depression (51).
• Patient preference for group versus individualized exercise, structured versus lifestyle PA, level of supervision desired, and attraction or aversion to specific exercise modalities must be considered to optimise behavioural change and long-term adherence.

### Gait training recommendations

Several trials investigating the effects of exercise interventions on gait ability (velocity and stability) in older individuals with frailty have yielded conflicting results. Whereas some studies showed improvements in gait ability after the physical training period (69, 70, 71), others found no improvement (69). Interestingly, the bulk of studies demonstrating improvements in gait ability utilised multicomponent exercise programmes (44, 69, 71, 72, 73), while others used only resistance exercises [74] or a combination of aerobic training and yoga (75).

Gait ability is a strong predictor of survival in older adults (76) and its maintenance should be prioritised in the oldest old. Aerobic exercises such as walking with changes in pace and direction, treadmill walking, step-ups, and stair climbing, among others are valuable modalities to achieve aerobic fitness adaptations and gait and mobility improvements. Weight-bearing aerobic activities that simulate real-life activities are preferable whenever possible. For those patients with severe arthritis or balance impairment, aquatic exercise, seated steppers, or recumbent cycles may be more tolerable alternatives. Generally, however, if someone cannot support their body weight independently, the initial priority should be resistance (strength, and power) training, as well as balance training, before moving to ambulation and other forms of weight-bearing aerobic exercise.

The duration of the exercise component may start at 5–10 minutes (or less) during the first weeks of training and progress to 20–30 minutes in the long term. The intensity of this exercise component is generally proportional to heart rate and can be increased from moderate to vigorous as fitness and confidence improve. If the heart rate can no longer function as an accurate measure of exercise intensity due to arrhythmias, beta-blockers or functioning pacemakers, clinicians can use perceived exertion scales instead. If cognitive impairment precludes the use of subjective perceived exertion, observer-rated perceived exertion (respiratory rate, ability to talk, sweating or facial expression) can be substituted.

### Resistance training recommendations

Evidence dating back to 1990 has demonstrated that high-intensity resistance training is feasible and effective even for older persons who are severely frail (77). To maximise adaptations of the musculoskeletal system and for time-efficiency, it is recommended that resistance training programmes be performed 2–3 times per week, starting with 1–2 sets and progressing to 2–3 sets of 8–12 repetitions. The resistance exercises should target major muscle groups of the upper and lower body involved in function and mobility. Routines may include both multi-joint exercises (leg press, chest press) and single muscle groups (triceps, knee extensors, hip abductors). Generally, 6–10 exercises may comprise the entire prescription, but this routine may be further broken down into sets of 3–4 exercises for novice trainees. A minimum of one day of rest is recommended between sessions involving the same muscle groups to allow time for the muscles to recover and for hypertrophic adaptations to eccentric muscle damage and repair to begin (78). To optimise the functional capacity of individuals with frailty, resistance training programmes should also include exercises in which daily activities are directly simulated, such as the sit-to-stand exercise or step ups.

There is no need to wait months before introducing high-intensity training for older adults with frailty. High-intensity training can be achieved by first measuring muscle strength with the one repetition maximum (1 RM) or the maximum amount of weight the individual can lift with good form in a single repetition of a muscle group. Exercise specialists can then use weights equivalent to 50, 60, 70, and then 80% of the 1 RM over the first four training days (i.e., 1 to 2 weeks). Indeed, different meta-analyses have shown that resistance training progressing to intensities ranging from 70 and 80% of 1RM promote greater strength gains than progressing to light (i.e., <50% of 1RM) and moderate (i.e., < 70% of 1RM) (79, 80). On each subsequent day of training, perceived exertion scales can be used to increase the load as tolerated by the patient, but striving to maintain the perceived exertion in the hard to very hard range (15 – 18 on the original Borg scale) (81). Perceived exertion can be rated subjectively by the individual and/or objectively by the trainer/caregiver when needed. This method has been used successfully in nursing home residents as old as 103 years of age, and in older patients 12 weeks after hip fracture surgery (68). Older adults with disabilities may need more direct supervision when performing muscle-strengthening activities than those who are more robust. Caution should be taken, especially during the first phase of an exercise program, because even a small injury may require weeks to recover and result in the exercise regimen being abandoned or started over. Whilst the absolute loads that can be lifted may be very low initially, even extreme frailty is not a contraindication to robust exercise. Indeed, frailty is one of the most crucial reasons to prescribe an exercise regimen.

### Power training recommendations

Power training is a specific type of muscle training in which both components of muscle power (force production and velocity) are targeted. It is considered a sub-category of resistance training. The main difference between power training and high-resistance strength training is, that strength refers to the ability to overcome resistance (involving high forces), while power refers to the ability to overcome resistance in the shortest period of time. Preserving muscle power output is critical to counteract the age-related decline of functional capacity and the earlier and more precipitous decline in muscle power compared to muscle strength (due to the preferential atrophy of type II fast twitch fibres with age) (82) underlies the emphasis on this modality. Deficits in muscle power are associated with disability in older men and even more so in women. Muscle power output and rate of force development are strongly associated with older persons' capacity to perform activities of daily living (83, 84, 85). In fact, research in healthy older adults has shown strong associations between performance in tests of functional capacity and muscle power output (83, 84, 85). More recently, investigators have found that training for muscle power and explosiveness are also associated with improved functional capacity and a reduction in the incidence of falls in oldest-old populations such as those who are frail and/or institutionalised (84, 85, 86). Muscle power training should be prescribed where possible to both healthy and older individuals with sarcopenia, frailty and other co-morbidities. The combination of power training with slow concentric velocity resistance training optimises functional ability, reduces the incidence of falls, improves muscle strength and power output, and stimulates muscle hypertrophy (27).

Optimal training regimens for maximising muscle power should be performed with the concentric (shortening) phase as fast as possible, followed by a controlled, slower eccentric (lengthening) phase, focused on the lower limbs (27, 87). Sets of explosive muscle actions can be performed alone (69, 88) or combined with traditional resistance training during the same session, but always avoiding concentric failure (87, 89, 90). Power is maximised at 30–45% loads for the upper extremities and at 60–70% of peak force capacity (one repetition maximum or 1 RM) for the lower extremity extensors (63, 91). In a dose-response study (63), peak muscle power improved similarly using light (20%), moderate (50%), or heavy (80%) resistances. There was a dose-response relationship between training intensity, muscle strength and endurance changes favouring high-intensity training (92). Therefore, using heavy loads during explosive resistance training may be a feasible strategy to achieve simultaneous improvements in muscle strength, power, and endurance in older adults. Nevertheless, it has also been shown that power training performed at low to moderate intensities (i.e., 40 – 60% of 1RM) induced marked increases in maximal strength, and power gains, functional capacity gains, muscle mass and quality, as well as reduce risk of falls in frail oldest old (27, 69, 87). Indeed, several of studies investigating the adaptations induced by the power training performed at moderate intensities (i.e., 60% of 1RM) (93) and high-speed muscle contraction (94) have observed superior adaptations in the functional capacity comparing traditional resistance training at higher loads. It should also be considered, that power training at low loads (during which velocity is much faster) poses a risk of meniscus or tendon injuries if undiagnosed degenerative changes are present, as is common in older adults (95, 96), and this is not recommended therefore.

Several studies have used standard free weights and weight machines for power training (69, 88, 89). Other studies used pneumatic resistance machines specifically designed for power training (92, 97, 98), resulting in similar neuromuscular and functional improvements [99]. As it is impossible to overcome resistance with momentum on such machines (as can be done by ‘swinging’ a free weight), they offer a theoretical and practical advantage. Plyometric training (e.g., jumping up onto platforms/boxes), which children and athletes have traditionally used for this purpose, may serve as an alternative training modality when power training machines are unavailable. However, arthritis and balance impairment may preclude the use of plyometrics by many older adults with frailty, who are precisely the individuals most in need of improvements in muscle power. Body weight as resistance (e.g., rising quickly from a chair) may serve as an alternative initial strategy. An individual could start with a slower execution and the assistance from another person. They can then progress until able to perform it alone and as fast as possible, with a slight jump at the end of the chair rise where feasible. This strategy may be easily implemented in hospital rooms, at home, or in aged care residences. Once body weight no longer serves as a sufficient source of overload for the capacity of the lower extremity muscles, additional resistance can be provided by machines or holding free weights as needed to ensure progression.

Importantly, special care should be taken with the execution of power training exercises to avoid musculoskeletal injuries. Clinicians should routinely screen patients for rotator cuff and meniscus tears before starting the explosive resistance training. Patients with osteoarthritis are particularly at risk for these types of injuries. These injuries may represent a significant barrier for older adults intending to perform power training. This challenge does not preclude the performance of a more traditional slow-velocity, high-intensity resistance training, or even isometric resistance training against an immovable object if any movement through the range of motion causes pain. As symptoms improve, the isometric contractions can progress to dynamic lifting through the pain-free range of motion until full range possible is achieved. During exacerbations of arthritis, this regression to isometric training, and then gradual resumption of dynamic and then power training movements can be utilised to prevent injury and optimise adaptations.

### Aerobic training recommendations

Ageing is associated with a decline in the cardiorespiratory capacity that is primarily associated with a decrease in the maximal heart output caused by a reduced maximum stroke volume and heart rate and changes in the oxygen arteriovenous difference (100). Aerobic training can counteract these age-associated phenomena, by inducing central and peripheral adaptations that enhance both maximal oxygen uptake (VO2max) and the ability of skeletal muscle to generate energy via oxidative metabolism (59, 101, 102, 103). The capacity to increase cardiorespiratory fitness is relatively preserved during ageing, although older adults rely more on increased stroke volume and blood pressure than on augmenting heart rate with maximal effort. There is a post-synaptic “β-adrenoceptor desensitization” responsible for a reduction in the autonomic modulation of the cardiac system, especially during physical exercise, which has been described for decades (104). Although stroke volume increases are similar in healthy young and older hearts, older adults demonstrate cardiac end-diastolic volume increases to augment stroke volume during exercise, rather than large increases in ejection fraction or heart rate. There is no evidence that maximal heart rate can be increased with exercise training, although the responses to sub-maximal exercise are attenuated, indicating improved tolerance (e.g., less dyspnoea and fatigue) as these sub-maximal loads now represent a lower fraction of peak exercise capacity. Therefore, aerobic capacity is an important component of physical fitness, and aerobic training should be part of the exercise routine for both fit and frail older adults (105). Such results have important clinical applications, since not only baseline aerobic fitness (which has a large genetic component), but also an increase of VO2peak over time in adulthood, is related to reduced risk of mortality (106) and many other chronic diseases.

However, older adults with severe functional declines may not be able to perform sufficient aerobic training to accrue benefits in the face of marked decrements in neuromuscular capacity (105). Indeed, it has been demonstrated that power and strength levels are positively associated with the cardiorespiratory capacity in elderly subjects (107), and loss of muscle mass has been reported to explain approximately 50% of the decline in peak aerobic capacity with age (108). In addition, targeting this loss of muscle function with PRT (without any aerobic exercise) has been shown to increase aerobic capacity by between 8–24% in older adults (58, 109). Based on such evidence, aerobic interventions in frail older individuals have sometimes included endurance training within multi-component exercise programs (72, 73, 110, 111). Thus, it may be necessary to strengthen the neuromuscular system, and improve balance, before initiating aerobic exercise to achieve adequate cardiovascular adaptations.

Aerobic exercises for older adults may include walking with changes in pace and direction, (71, 72) treadmill walking (73, 111) step-ups, stair climbing, and stationary cycling (73), dancing or aquatic exercise. The choice of modality should depend on individual preference, accessibility, cognitive and physical comorbidities, and specific musculoskeletal issues. For example, the feasibility, weight bearing nature, and functional relevance of walking and its variants suggests this as an ideal modality for many people. However, severe balance impairment, peripheral neuropathy, neuromuscular disease, orthostatic hypotension, or lower extremity arthritis may suggest recumbent cycling or aqua aerobics as more realistic alternatives in such cases. Arm ergometry may serve as an aerobic substitute for those with stroke, leg ulcers, amputations, or other conditions precluding use of lower extremities temporarily or permanently. In all cases, sequencing should be considered, and introduction of strength/power training, then balance exercise, and then finally aerobic training in that order may be the key to success and safety.

The endurance exercises may start with a duration of 5–10 min in the first weeks of training, progressing to 15–30 min for the remainder of the program, with frequencies from 3 to 7 days per week. There is no reason to rest a day between sessions, and it is possible to break sessions into small segments of a few minutes throughout the day without diluting benefits.

Improvements in aerobic capacity (and most other health outcomes) are best achieved with moderate- to- vigorous-intensity aerobic exercise (MICT), with the greatest effects for fitness and some health outcomes seen with high-intensity interval training (HIIT, 85–95% peak heart rate for 1–4 minutes intervals) (56, 112).

In some older adults, heart rate is not an adequate index of aerobic intensity, due to the presence of atrial fibrillation, pacemakers, or beta-blockers. Other methods for controlling the exercise intensity are thus required, such as the use of rating of perceived exertion scales (e.g., the Borg Scale) (81) on which intensities of 12–14/20 appear to be well tolerated and correspond to moderate-intensity aerobic exercise.

In order to continue to adapt to aerobic exercise, progressive increases in the intensity of training are needed, as with all other exercise modalities. In older adults, this can be difficult when gait and balance disorders or osteoarthritis preclude typical progression to higher impact activities, such as jogging or running. Examples of feasible ways of increasing aerobic intensity without increasing impact on joints with arthritic changes or osteoporotic fragility fracture risk include:

• Walking—add small weights around wrists, swing arms; use race walking style, add inclines, hills, or stairs; carry weighted backpack or waist belt; push a wheelchair or stroller (with someone in it).
• Cycling—increase pedalling speed, increase resistance to pedals, add hills.
• Water activities—use arms and legs in strokes, add resistive equipment for water; increase pace.
• Tennis—convert from doubles to singles game.
• Golf—carry clubs, eliminate golf cart.
• Dance—increase pace of movements, add more arm and leg movements.

### Balance training recommendations

Due to medical conditions, many older adults require balance training before aerobic exercise/gait retraining can be adequately undertaken. A potential challenge during the performance of balance training is the possibility of an accidental fall (45). Therefore, a common-sense approach is to practice the most challenging posture or movement possible without falling in a safe environment (e.g., standing on one leg without hand support). Once the exercise level is ‘mastered’, the individual can then progress to the next-harder level, for example with eyes closed. This is a similar principle to the one applied during progressive resistance training: as soon as a load no longer feels ‘hard’ to lift on the perceived exertion scale, it can then be increased to ensure continuous, optimum adaptation. It is important to consider that, even in frail individuals, improvements in balance performance are stimulated when balance exercises are correctly applied (105). An in-depth discussion of balance training is beyond the scope of this paper. The most effective principles of balance training are shown in Table 1.

### Multicomponent training

Multicomponent training programmes (generally inclusive of various combinations of strength, power, gait, balance, and functional training programmes) should include gradual increases in the volume, intensity, and complexity of the individual exercises. This exercise training modality may also be prescribed to the most vulnerable populations, including acutely ill, hospitalised older patients (113) or institutionalised older adults (44, 69, 114).

### Multicomponent Training in Dementia

Research studies in older adults with cognitive impairment show the feasibility and efficacy of multicomponent exercise interventions combined with cognitive training, nutritional strategies, and social enrichment (115, 116, 117, 118). However, it is not clear whether multicomponent interventions are more effective than single-exercise protocols (119, 120, 121). Supervision is vital, and there is no evidence that low-intensity, minimally progressive multimodal exercise improve cognitive outcomes in patients with mild (122) or moderate dementia (123). Additional recommendations include consideration of dementia-related behavioural issues, and communication challenges. Simple instructions rather than complicated verbal instructions, constant reassurance, and the use of mirror techniques may help the patient achieve meaningful progress during training sessions. Creating a respectful, mindful and empathetic training atmosphere for individuals with cognitive impairment may promote their participation and adherence (44, 69, 114). Examples of evidence-based instructions for mindful caregiving combined with home-based, progressively intense resistance and balance training for the dementia dyad (caregiver and loved one with dementia) are available (http://www.strongmindshomecare.org).

The VIVIFRAIL Multicomponent Physical Exercise Program to Prevent Frailty and the Risk of Falls represents an excellent example of an evidence-based program. The Vivifrail physical exercise guide, (http://vivifrail.com/ resources/) consists of lower-limb (squats rising from a chair, leg press, and bilateral knee extension), upper body (seated bench press), balance and gait re-training (e.g., semi-tandem line walking, single-leg standing, stepping practice, walking with small obstacles, proprioceptive exercises on unstable surfaces such as foam pads sequence, weight transfer from 1 leg to the other) exercises. Vivifrail has individual prescription passports for older adults which can be implemented during unsupervised sessions. The exercise recommendations are tailored to the older person's functional capacity level (severe limitation, moderate limitation and slight limitation as evaluated by the Short Physical Performance Battery (SPPB) and a walking speed test) and risk of falling (http://www.vivifrail.com) (114, 124, 125). Recent evidence shows that intermittent strategies such as 4 weeks of supervised VIVIFRAIL exercise 3 times yearly with no more than 14 weeks of inactivity between exercise periods appears as an efficient solution to the global challenge of maintaining functional capacity and can even improve frailty in vulnerable institutionalized older adults (114, 126).

## Safety of long-term physical activity and exercise interventions in Older Adults

Systematic reviews have reported that long-term (>1 year) physical exercise interventions in older adults do not increase the risk of dropouts due to health issues, mortality, hospitalisation or fracture compared with a usual care group (127, 128). In contrast, these types of interventions are associated with a lower risk of falls and fall-induced injuries, while improving muscle strength, balance, physical function and cognition (128). This evidence suggests that long-term physical exercise is less harmful for older adults than maintaining low usual activity levels. Similarly, epidemiological data show that although the risk of myocardial infarction (MI) is greater during exercise than at rest, the overall risk of an MI is 50% lower in those who are regularly active, and is 50-fold lower during an acute bout of exercise in active adults compared to those who do not engage in regular exercise (129). Therefore, long-term physical exercise is safe and effective in older adults, and its benefits appear to be independent of the participants' age, physical function or cognitive status at baseline (128). Sedentariness is the lethal condition.

The type and frequency of physical exercise training (i.e., resistance training, aerobic MICT or HIIT), age, cognition and physical function level do not influence the effects of exercise on attrition rates secondary to medical problems and mortality (128). Clinicians have been reluctant to prescribe resistance training to older adults despite evidence that a properly designed resistance exercise program may, in fact, reduce the overall risk of adverse events by substituting potentially inappropriate medications (43, 128). Both research and clinical experience indicate that resistance training is safe for healthy older adults (130), frail (physiologically vulnerable) older adults (131, 132), and individuals with multiple chronic diseases (43). Engaging in resistance training reduces the risk of incident diabetes (133, 134) and cardiovascular disease (135), and also significantly reduced the risk of all-cause and cancer mortality (136, 137). Improvements in cognition (120, 121), diabetes control (138, 139), cardiac rehabilitation outcomes (140), renal failure (141), COPD (142), arthritis, Parkinson's Disease, stroke, and many other pre-existing diseases have also been shown improve after the application of evidence-based resistance training.

High-intensity interval training (HIIT) has now been incorporated into some public health recommendations as a form of aerobic exercise (143). It may offer benefits as compared to moderate-intensity exercise of longer duration in terms of efficiency, shorter duration, and better tolerance in those individuals with chronic fatigue, lower extremity arthritis, claudication, and/or low thresholds for dyspnoea due to lung diseases. However, reports on the efficacy and safety of exercise come primarily from studies in healthy and cardiovascular cohorts (144). The feasibility of exercise in older adults with frailty and multimorbidity remains to be established. More recently, all-cause mortality and severe events including the onset or worsening of cardiovascular disease, the occurrence of cardiovascular events, injuries and fractures were examined in older adults. Participants were randomised to two sessions weekly of High-intensity interval training at about 90% of peak heart rate (HIIT), moderate-intensity continuous training at about 70% of peak heart rate (MICT), or to follow the national guidelines for physical activity; all for five years [16]. This study observed a non-significant 1.7% absolute risk reduction in all-cause mortality in the HIIT group compared with recommended PA levels (Control), and a trend for lower all-cause mortality after HIIT compared with MICT. Ongoing studies in mild cognitive impairment, diabetes, and many other conditions will extend the evidence base and data on feasibility of application and efficacy in other clinical cohorts. Conditions where prolonged weight-bearing forms of aerobic exercise may be problematic (peripheral neuropathy, peripheral vascular disease, severe arthritis, obesity, or low thresholds for ischaemia or desaturation or fatigue) are particularly relevant in this regard.

## Role of physical activity and exercise on bone health, adipose tissue, muscle mass, and maximal strength and power

Many studies suggest that habitual engagement in PA/ exercise can markedly attenuate most decrements in exercise capacity that would otherwise occur with ageing. In the last few years, there has been accumulating evidence from well-designed studies supporting the benefit of PA for bone health, increase of muscle mass and strength/power, and reduction of adipose tissue (Table 2).

**Table 2:** Exercise recommendations targeting optimal body composition for older adults

**Exercise recommendations**	**Decreased adipose tissue mass and visceral/centraldeposition**	**Increased muscle mass and function**	**Increased bone mass and density and reduced fracture risk**
Modality	• Aerobic or resistance training	• Resistance training	• Resistance training • High-impact activities (e.g. jumping using weighted vest during exercise) if tolerated by joints. Not recommended for people with vertebral osteoporosis • Balance training
Frequency	• Aerobic: 3–7 days/week • Resistance: 3 days/week	• 3 days/week	• Resistance training: 3 days/week • Balance training: up to 7 days/week
Volume	• Aerobic: 30–60 min/session • Resistance: 2–3 sets of 8–10 repetitions of 6–8 muscle groups	• 2–3 sets of 8–10 repetitions of 6–8 muscle groups	• 2–3 sets of 8–10 repetitions of 6–8 muscle groups • 50 jumps per session for high impact^a^ • 2–3 repetitions of 5–10 different static and dynamic balance postures
Intensity	• Aerobic: 60–75% of maximum exercise capacity (VO2 max or maximum heart rate) or 13–14 on the Borg Scale of perceived exertion • HIIT training: 85–95% peak heart rate; 1 to 4 intervals of 4 min, 3 days/week • Resistance: 70–80% of maximum strength (one repetition maximum) exertion	• 70–80% of maximum capacity (one repetition maximum)	• 70–80% of maximum capacity (one repetition maximum) as load • 5–10% of body weight in vest during jumps; jumps or steps of progressive height • Practice the most difficult balance posture not yet mastered


a. Thus far proven only in premenopausal women and adolescents or when combined with resistance training/multi-modality exercise in older adults; HIIT: High-intensity interval training

### Bone health, physical activity, exercise, and fracture risk

Bone mass begins to decrease well before menopause in women (as early as the 20s in the femur of sedentary women). It accelerates in the perimenopausal years, with continued declines into old age. Similar patterns are seen in men, without the acceleration related to loss of ovarian function seen in women. As with losses of muscle tissue and function (sarcopenia), many factors related to genetics, lifestyle, nutrition, disease, and medications may predict bone density at a given age. Epidemiologic studies have shown that a 10% increase in peak bone mass at the population level reduces the risk of fracture later in life by 50%. The accumulation of peak bone mass and bone strength in childhood and adolescence is important in order to reduce the osteoporosic fracture risk in later life (145).

Mechanical loading of the skeleton generally leads to favourable site-specific changes in bone density, morphology, or strength, whereas unloading (in the form of bed rest, immobilisation, casting, spinal cord injury, or space travel) produces rapid and sometimes dramatic resorption of bone, increased biochemical markers of bone turnover, and therefore, changes in morphology such as increased osteoclast surfaces, and increased susceptibility to fracture. Significantly higher bone density has been observed in athletic cohorts with the effects depending on the type, intensity, and duration of exercise training and the characteristics of the athletes [146]. Exceptions include non-weight-bearing activities (i.e., swimming, cycling), amenorrhoeic women or competitive distance runners, whose bone density appears similar to or lower than that of controls.

The incidence of hip fractures is 30–50% lower in older adults with a history of higher PA levels than age-matched, less active individuals. In the prospective Epidemiology of Osteoporosis (EPIDOS) study of 6901 white women ≥ 75 years old followed for 3.6 years (147), low PA levels increased the risk for proximal humerus fracture by more than two-fold.

Significant changes of bone health in the femur, lumbar spine, and radius have been seen following high-impact aerobic training, resistance training, and combined aerobic and resistive exercise programmes. The effectiveness of isolated high-impact training (jumping, skipping, heel drops) seen in younger women, has not been replicated in studies of postmenopausal women (148).

#### Optimal exercise modality and intensity for bone health

The predominant exercise training factor that influences bone adaptation is the intensity and novelty of the load. Studies in animals on the effects of mechanical loading show that bone is most sensitive to short periods of loading characterised by unusual strain distribution, high strain magnitudes, and a rapid loading rate (149).

Kohrt et al. (150) found that aerobic activities with high ground-reaction forces (walking, jogging, stair climbing) and exercises with high joint-reaction forces (weightlifting, rowing) in older women significantly increased the bone mineral density (BMD) of the whole body, lumbar spine, and Ward's triangle. In contrast, only the ground-reaction group increased BMD at the femoral neck (150). The femoral-neck BMD increased only in the ground-reaction forces group while the lean mass and muscle strength increased only in the weight-lifting group necessary for gait improvement and fall reduction. These studies suggest that both types of exercises are equally important in falls and fracture prevention. In another study, progressive strength training in postmenopausal women resulted in significant increases in total and intertrochanteric BMD after two years (151). In general, as compared with aerobic training, resistance training in older adults is more favourable due to its broader benefits on muscle, bone, balance, and fall risk. If aerobic training is used, however, activities that are weight-bearing and higher impact have greater efficacy for bone health than non-weight-bearing or low-impact aerobics activities (152).

It is essential to prescribe the optimal modality of exercise and the relative intensity, as the skeletal adaptation is critically linked to the intensity of the loading (whether due to increased amount of weight lifted during resistance training or higher ground-reaction forces during aerobic/jumping activities). Multimodal exercise programme over 12 months (high-intensity resistance training and a weight-bearing circuit of moderate-impact activities including walking/jogging, skipping, hopping, and stair climbing/stepping with weighted vests) resulted in significant BMD improvements at the femoral trochanter. These results were linearly related to total weight lifted and exercise specific weight lifted (e.g., leg press, squat, and military press exercises), but not to the volume or quality of the non-resistance training components of the programme (153). Moreover, multicomponent exercise training that included progressive resistance exercise of moderate-vigorous intensity prevented increase in bone turnover and attenuated decrease in hip BMD induced by weight loss in frail, older adults with obesity (154).

Overall, most studies demonstrating the efficacy of exercise on BMD have been conducted in women between 50 and 70 years of age, and it is not yet known if the efficacy would be similar in women over 80 with multiple comorbidities, who have often been excluded from such trials (51, 155, 156). For example, a randomized clinical trial (RCT) of 90 men and 90 women aged 65–74 comparing Tai Chi, resistance exercise and control three times a week for 12 months on BMD, muscle strength, balance and flexibility in community-dwelling people showed modest effects only which may not translate into better clinical outcomes, although adherence rate was high (157). However, the most recent studies suggest that optimal adaptations continue to accrue with high-intensity resistance and power training in older adults (158).

### Adipose tissue, physical activity and exercise

Ageing is associated with changes in body composition including increased visceral adipose tissue, redistribution of visceral adipose tissue from the periphery to a central distribution and deposition of ectopic adipose tissue. All of these changes are risk factors for pathology including; osteoarthritis, cardiovascular disease, gall bladder disease, type 2 diabetes, breast, colon, and endometrial cancer; hypertension, stroke, dementia and reduction in vascularization and hypoxia, increased fibrosis and senescent cell accumulation (159). Reductions in visceral fat have been shown to improve glucose tolerance and insulin sensitivity in those with and without diabetes. Changes in trunk fat correlate with improved glycaemic control in type 2 diabetes (139, 160). Hence the potential for exercise to favourably impact the accretion and distribution of adipose tissue with age is critical to chronic disease accumulation. In this section, we will review the significance of the effects of exercise on the reduction of disease burden associated with reducing adipose tissue in older adults.

#### Experimental studies of the influence of physical activity on abdominal fat

Evidence from well-designed studies support the benefits of PA in reducing total abdominal fat. Most studies have included middle-aged to older populations with higher abdominal and visceral fat accumulations than that found in younger adults. These studies were more likely to demonstrate a greater magnitude of change than in subjects with lower amounts of abdominal fat mass at baseline (161). Furthermore, the potential for PA to attenuate the gains in visceral fat is evident in obese individuals as early as during childhood.

Decreases in both total adipose tissue accumulation and its central and visceral deposition are achievable by both aerobic and resistance training. However, reductions in total body adiposity are more rapid when combined with energy-restricted diets or when performing very large volumes of exercise (i.e., 7 hours per week resulting in high energy expenditure), both of which support a negative energy balance. However, these intensive interventions are often not attainable in clinical practice, especially in older adults. Preferential visceral fat mobilisation is often seen in response to exercise and dietary intervention, which means that small amounts of total body weight or fat mass (5%) reduction may be associated with substantial changes in visceral fat (25% or more). These changes in adipose tissue will have important metabolic implications for preventing or treating the insulin resistance syndrome and type 2 diabetes (162).

The combination of exercise and diet are the most effective non-surgical treatment for obesity. International consensus panels uniformly advocate this approach. The advantages of adding exercise to diet include more significant weight loss, preservation of fat-free mass, preservation of resting metabolic rate (when resistance training is included), improved fitness levels, correction of metabolic abnormalities associated with visceral obesity, improved fitness and co-morbid diseases, and better long-term adherence to dietary modifications, resulting in sustained weight maintenance. Therefore, exercise plus diet appear to represent an optimal initial evidence-based treatment for obesity in individuals of all ages.

#### Relationship between exercise intensity and changes in body fat

In general, weight loss parallels energy expenditure via exercise, whether achieved by more significant volumes, intensities, or durations of the exercise prescription. There is no evidence from well-designed studies demonstrating that low-intensity exercise is effective in reducing abdominal fat. Most robustly designed studies have used moderate- to high-intensity aerobic interventions. An overall higher-intensity stimulus can be delivered via intermittent intensities with resistance or interval training. This exercise prescription may be effective and better tolerated by ‘at-risk’ populations than sustained, moderate or intense exercise due to lower extremity osteoarthritis, restrictive lung disease or claudication, for example, which may accompany obesity.

Furthermore, in a recent meta-analysis of studies comparing body composition changes between interval training and moderate-intensity continuous training, it has been suggested that intensity of effort during endurance exercise has minimal influence on longitudinal changes in fat mass and lean mass (163). These studies underscore the importance of overall exercise volume (resulting in higher energy expenditure) to facilitate fat mass loss. However, the amount of exercise required to achieve practically meaningful changes in fat mass (∼100 minutes/day) is unrealistic for the majority of individuals who are overweight/obese, and thus of limited practical relevance. Therefore, dietary prescription plays a key role to create an energy deficit and facilitate fat mass loss (164). However, exercise may help to preserve lean mass (both muscle and bone) and functional performance during periods of energy restriction (165) and should be considered as an important supplement to nutritional approaches for those who endeavour to reduce adiposity.

#### Relationship between exercise modality and changes in body fat

There is some evidence that aerobic training may be better than resistance training for reducing abdominal fat (166). However, at doses resulting in a sustained negative energy balance for several months, both resistance and aerobic exercise generally result in significant reductions in fat mass when sensitive measurement techniques (generally not anthropometrics) are used. Resistance exercise may be more suitable as a fat-reduction strategy for older obese individuals with cardiovascular disease, arthritis, osteoporosis, peripheral vascular disease, or mobility disorders, who may not tolerate moderate- to high-intensity aerobic training or may need the added benefits of resistance training for maintenance of muscle and bone mass. Importantly, energy restriction results in significant losses of muscle and bone. The addition of resistance training to hypocaloric dieting has been shown to prevent such adverse changes in body composition (167), not attained with aerobic exercise alone. The combination of aerobic and resistance training has demonstrated superiority in reducing trunk fat in older men than aerobic training alone [168, 169, 170]. More well-designed studies are needed, particularly in overweight/obese older adults, to explore the relative benefits of these modes of exercise for optimising body composition.

### Role of physical activity and exercise in preserving muscle mass with age

In contrast to changes in fat and bone, an increase in muscle mass is achievable to a significant degree only with progressive resistance training or weight gain from extra energy and protein consumption. Accretion of lean tissue with exercise has a potentially beneficial effect in preventing diabetes and metabolic syndrome (171), functional dependency, falls, and fractures, treatment of chronic diseases, disabilities, which are often accompanied by disuse, catabolism, and sarcopenia. For patients with type 2 diabetes mellitus, there are potential advantages to both minimising fat and maximising muscle tissue since these compartments have opposite and likely independent effects on insulin resistance in older adults. Resistance exercise coupled with leucine-enriched essential amino acid supplements or whey protein food supplements (when the diet is inadequate in energy and/or protein) are recommended to treat sarcopenia (172), although recent reviews have not found additional benefit for protein above resistance training alone in older adults who are not malnourished (173). There is evidence from various epidemiological and experimental studies that muscle weakness, decreased muscle mass, reduced activation of glycogen synthase, and alterations in numbers of glycolytic skeletal muscle fibres are related to, and may precede, insulin resistance, glucose intolerance, and type 2 diabetes expression (174, 175).

#### Exercise to maintain or increase muscle mass and strength

A properly designed resistance training programme can counteract or ameliorate the age-related changes in contractile function, atrophy, and morphology of ageing human skeletal muscle (47). Appropriate progressive resistance training programmes of 3–6 months in duration can increase muscle strength by an average of 40–150%, depending on the person's characteristics and intensity of the programme and to increase total body lean mass by 1–3 kg or muscle fibre area by 10–30% (176, 177). Exercise training reduces frailty in older adults by suppressing muscle inflammation and promoting anabolism which leads to an increase in muscle protein synthesis rate (73, 178, 179), in addition to its benefits on other contributors to frailty such as anorexia, depression, self-efficacy, cognitive dysfunction, gait and balance disorders, and cardiometabolic profile (180, 181, 182). Thus, even if some of the neural control of muscle and the remaining absolute number of motor units are not affected by exercise, the adaptation to muscle loading, even in very old age, causes neural, metabolic, and structural changes in muscle, which can compensate for the strength losses and, to some degree, age-related atrophy (183). Generally, strength gains after exercise far exceed, and are not directly correlated with, muscle size changes due to the importance of neural adaptation, particularly early in this process.

High-intensity resistance training is also more beneficial than low-intensity training for simultaneously maximising muscle and bone mass and strength, and treating gait disorders, functional impairments, and disability, making it ideal as a multiple risk factor intervention strategy for injurious falls prevention in osteopenic/osteoporotic adults.

#### Predictors of muscle hypertrophy after exercise

There is mixed evidence about whether there are significant sex differences in the functional or hypertrophic response to resistance training in older adults, heavily influenced by the presentation of the results in either and absolute or relative context (184). Some studies have found women to have smaller gains in muscle strength, power, or hypertrophic response to training, whereas others have found no differences or even greater relative gains in women. It is likely that differences in training regimens (particularly related to intensity) and measurement techniques used to assess muscle mass, cross-sectional area or volume may explain some of these discrepant results. Malnutrition, impaired protein synthesis rates, inflammatory cytokines, low self-efficacy and depression are other factors that have been identified as barriers to engagement in (185), and/or robust adaptations to resistance training in older adults. The blunted response to exercise and nutritional stimulation of muscle protein synthesis has been termed “anabolic resistance” (186, 187). Furthermore, the role of genetic and epigenetic influences on adoption and adaptation are still under investigation (188).

### Role of physical activity and exercise in primary, secondary and tertiary Disease Prevention

Physical activity and structured exercise can lessen the burden of comorbidity, disability, and premature death caused by incident disease and is beneficial for primary, secondary, and tertiary prevention. Habitual PA patterns may be influenced by ageing and genotype as well as acute and chronic disease accumulation and ecological factors (see Figure 1). Activity engagement, in turn, may affect physiological capacity, psychological health, dietary intake, adverse behaviours or risk factors for chronic disease (see Figure 2). These are all potential bi-directional pathways by which exercise may ultimately influence the prevalence of chronic disease in a population.

Although appropriate levels of PA may optimise risk factor profiles, the presence of risk factors may lead to reduced PA and thus heightened risk of disease. For example, inactivity may lead to loss of muscle mass, followed by muscle weakness and further restriction in activity levels, subsequently contributing to the development of osteopenia and gait abnormalities, and potentially a high risk for falls and hip fracture.

Epidemiological data suggest that exercise habits in middle-aged sedentary adults with low fitness markedly the risk of cardiovascular mortality (189), suggesting that exercise initiated at middle age or beyond may be as important as when started at a younger age to reduce mortality. Experimental data are available demonstrating the prevention of some diseases with exercise (e.g., secondary cardiovascular events, diabetes mellitus, and osteoporotic fracture). However, robust RCTs are not yet available to confirm reported epidemiological risk reduction for other conditions (e.g., renal failure, stroke, dementia, depression). Based on findings from the Finnish Diabetes Study (171) and the Diabetes Prevention Program(DPP), among others (190, 191), diabetes is clearly preventable in high-risk obese adults with impaired glucose tolerance through diet and exercise intervention (192). The DPP participants randomly assigned to the intensive lifestyle intervention of diet and exercise reduced their risk of incident type 2 diabetes by 58% at 3 years compared to the control group (lifestyle advice only), and the lifestyle intervention was significantly better than metformin prescription (193). Of particular interest is the finding that those over the age of 60 showed the best response with a 71% reduction in incident diabetes during this time frame. Metformin was no better than the control condition in this older age group. It is noteworthy that whilst metformin is often subsidised by some governments and health insurance plans for diabetes prevention in older adults (despite its lack of efficacy in this cohort), long-term lifestyle interventions are not reimbursed.

The major diseases and syndromes for which exercise may be beneficial as a preventive strategy or prevention of progressive disease (secondary and tertiary prevention) are listed in Table 3, along with the postulated mechanisms of exercise benefit and the specific modality of exercise most relevant for these outcomes.

**Table 3:** Role of Exercise in Primary, Secondary and Tertiary Disease Prevention

**Disease**	**Postulated mechanisms of exercise effect on disease prevention**	**Considerations for the prescription for secondary and tertiary prevention (disease expression and progression)**	**Recommended exercise modality**
Arthritis	• Decreased body weight • Maintenance of cartilage integrity • Maintenance of muscle and tendon strength	• Low impact • Sufficient volume to achieve a healthy weight if obese	• Aerobic exercise • Resistance exercise^a^
Cancer (breast, colon, prostate)	• Decreased body fat • Decreased oestrogen levels • Altered dietary intake • Decrease in gastrointestinal transit time • Increased prostaglandin F2	• Resistance training with dietary intervention may offset myopathy and reduce prevalence of cancer cachexia	• Aerobic exercise • Resistance exercise^a^
Chronic obstructive pulmonary disease	• Increased adherence to smoking cessation, dietary behaviours • Increased muscle mass • Improved lung function	• Resistance training may be more tolerable in severe disease, combined effects complementary if feasible • Time exercise sessions to coincide with bronchodilator medication peak. • Use oxygen during exercise as needed	• Aerobic exercise^a^ • Resistance exercise^a^
Chronic renal failure	• Reduced risk of hypertension • Reduced risk of type 2 diabetes mellitus	• Exercise reduces cardiovascular and metabolic risk factors, improves depression • Resistance training offsets myopathy of chronic renal failure	• Aerobic exercise • Resistance exercise^a^
Congestive heart failure	• Decreased risk of ischaemic heart disease • Decreased risk of hypertension • Decreased risk of type 2 diabetes mellitus	• Improves cardiovascular function and contractility • Improves hypertension and lipid profile	• Aerobic exercise • Resistance exercise^a^
Coronary artery disease	• Decreased blood pressure • Decreased LDL cholesterol • Increased HDL cholesterol • Decreased fibrinogen • Decreased total body fat, visceral fat • Decreased insulin resistance, hyperinsulinaemia • Decreased cortisol levels, inflammatory cytokines • Increased adherence to smoking cessation, dietary behaviours • Decreased depression, anxiety • Improved endothelial cell function	• Complementary effects on exercise capacity and metabolic profile from combined exercise modalities • Resistance training may be more tolerable if the ischaemic threshold is very low due to lower heart rate response to training	• Aerobic exercise • Resistance exercise
Dementia	• Improved cerebral blood flow • Increased neurotrophic factors in CNS • Hippocampal neurogenesis • Anabolic hormones • Prevention of diabetes/insulin resistance • Prevention of stroke • Prevention of hypertension • Prevention and treatment of depression	• Exercise under supervision if cognition is moderately to severely impaired • Avoidance of head trauma during exercise is critical	• Aerobic exercise • Resistance exercise^a^
Depression	• Increased self-efficacy, mastery • Internalised locus of control • Decreased anxiety • Improved sleep • Increased self-esteem • Increased social engagement, decreased isolation • Decreased need for drugs associated with depression (beta blockers, alpha blockers, sedative hypnotics) • Decreased body fat, improved body image	• High-intensity resistance training and adequate volumes of aerobic exercise are more efficacious than low-intensity/low-volume exercise in major depression	• Aerobic exercise • Resistance exercise^a^ • Yoga/other mind-body exercise^a^
Osteoporosis / Osteoporotic fracture	• Increased bone density • Increased tensile strength • Increased muscle mass • Improved gait stability and balance • Improved nutritional intake (energy, protein, calcium, vitamin D) • Reduced fear of falling, improved self-efficacy • Increased overall activity levels, mobility • Decreased need for drugs associated with postural hypotension, falls, hip fractures (antidepressants, antihypertensives, sedative-hypnotics)	• High-impact, high-velocity activity (e.g., jumping) is potent if tolerable; avoid if osteoarthritis is present. • Resistance training effects are local to muscles contracted. • Balance training should be added to prevent falls and must be challenging	• High-impact exercise^a^ • Resistance exercise^a^
Peripheral vascular disease	• Prevention of hypertension • Prevention of diabetes • Improved lipid profile • Assistance in smoking cessation • Reduction in adiposity/visceral adiposity	• Vascular effect is systemic; upper limb ergometry may be substituted for leg exercise if necessary • Resistance training has a similar effect on claudication as aerobic exercise • Low-intensity resistance training is ineffective. • Exercise only to the onset /early phase of claudication; rest and repeat • Avoid trauma to skin or feet; high impact training	• Aerobic exercise • Resistance exercise^a^
Stroke	• Decreased obesity • Decreased cholesterol • Prevention of diabetes • Prevention of hypertension	• Start with resistance and balance training until ambulation is safe • Cognitive impairment may require close supervision • Avoid Valsalva and breath holding to minimise hemodynamic excursions	• Aerobic exercise • Resistance exercise^a^ • Gait and balance exercise; mobility training^a^
Type 2 Diabetes Mellitus	• Improved insulin sensitivity • Increased GLUT-4 protein and translocation to membrane sites • Reduced visceral fat mass • Decreased cortisol response to stress • Improved lipid profile • Decreased blood pressure • Increased muscle mass	• Exercise at least every 48 hours to optimise glucose regulation • May need to avoid impact exercises if peripheral neuropathy present • Monitor blood glucose before and after exercise if not well-controlled	• Aerobic exercise • Resistance exercise (combined with diet and aerobic exercise)
Venous stasis disease	• Increased muscle mass • Decreased adiposity	• Local muscle contractions stimulate the return of fluid via the lymphatic system • Utilise lower body training; elevate legs when possible • Avoid trauma to skin	• Aerobic exercise^a^ • Resistance exercise^a^


a. Indicates that the modality of exercise has been shown to affect the postulated mechanistic factors and/or improve established disease expression or outcomes but has not yet been reported to prevent the distal disease outcome in either epidemiological or clinical studies.

### Role of physical activity and exercise in the secondary and tertiary Prevention

Exercise is particularly good at targeting syndromes of disuse and slowing down the trajectory of decline, especially in conditions such as Parkinson's disease, chronic obstructive pulmonary disease, and cardiometabolic diseases. Some disease-related pathophysiological abnormalities are specifically targeted by exercise, making it a valuable adjunct to standard care. Muscle-derived myokines are responsible for many of the beneficial effects of exercise by promoting a healthy anti-inflammatory and anabolic milieu (42). For example, adipose tissues are associated with inflammaging (194). Losses of visceral fat achieved through resistance or aerobic training improve insulin resistance and complement dietary and pharmacological management benefits in older adults with type 2 diabetes and central obesity (43). Regular exercise induces antiatherogenic adaptations in vascular function and structure, irrespective of traditional cardiovascular disease (CVD) risk factors (42). Similarly, exercises designed to stimulate skeletal muscle hypertrophy in congestive heart failure counteract the catabolic effects of circulating cytokines not achieved with cardiac medications alone (195). Lower-extremity exercises in individuals with arthritis improve joint stability, mobility, functional status, quality of life and pain (196).

It is not possible in this statement to review every disease in which exercise has beneficial effects. Therefore, we will use type 2 diabetes as a prototypical example of the disorders outlined in Table 3.

### Exercise in type 2 diabetes

Targeting glycaemic control without simultaneously addressing central obesity and sedentary lifestyle may hasten the emergence of disease complications and add to the burden of polypharmacy in susceptible individuals with insulin resistance. Weight loss by diet without engaging resistance exercise in older adults with obesity leads to losses of lean tissue (muscle and bone) which could exacerbate age-related sarcopenia and osteopenia (197).

Many consensus statements (34, 198) and position statements (199) recommend moderate- or high-intensity aerobic exercise of 3–4 h per week. This exercise frequency and intensity may improve insulin sensitivity and glucose homeostasis, assist in attaining or maintaining lower body weight, reduce visceral fat depots, modestly improve blood pressure and lipids, and lower the risk of cardiovascular morbidity and mortality. However, the clinical management of the obese individual with diabetes is often complicated by the presence of multiple other comorbid conditions that may impede adherence with both diet and aerobic exercise recommendations. Lack of adherence to exercise may affect quality of life, cognitive impairment, osteoarthritis, ischaemic heart disease, hypertension, hyperlipidaemia, peripheral vascular disease, sleep apnoea, peripheral neuropathy or stroke affecting gait and balance, renal disease, postural hypotension, bladder dysfunction, retinal disease, and depression (43). Such disease clustering makes the adherence to consensus statements and clinical guidelines difficult or even impossible as the performance of aerobic exercise at the volumes and/ or intensities shown to produce metabolic benefits becomes unrealistic for these individuals.

An alternative approach to aerobic exercise recommendations for adults with diabetes is the use of progressive resistance training. The specific benefits of resistance training in older adults with diabetes include its ability to combat age and diabetes-related sarcopenia, prevent loss of muscle and bone mass, reduce resting metabolic rate accompanying hypocaloric dieting, increase glucose uptake and storage in skeletal muscle, reduce visceral fat depots, reduce C-reactive protein, and provide beneficial effects on resting blood pressure, functional status, mobility, sleep, peripheral vascular disease, peripheral neuropathy, cognitive function, and depressive symptoms, among others (200, 201, 202). The effects on muscle mass are unique to high-intensity resistance training and clearly distinguishable from aerobic exercise. Exercise may also help prevent dementia in older adults with diabetes, although more evidence is needed (203]). For this reason, current recommendations include aerobic and resistance training as well as dietary modification for type 2 diabetes.

Type 2 diabetes substantively increases the risk of frailty and disability, with two to three times increased odds of disability across all functional groups reported from the NHANES cohort [204], and many others. In older adults with coexisting frailty, diabetes, and functional decline, evidence from a large RCT shows functional benefits with a combined approach of progressive resistance training, nutritional education, and readaptation of the clinical targets for glycosylated haemoglobin and blood pressure. In this study the benefits were evident early (8 weeks after starting the physical exercise program) and persisted over 12–24 months [205, 206]. There is also evidence about the benefit a multicomponent exercise programme consisting of resistance, endurance, balance, and gait retraining should be employed to increase functional capacity, quality of life, and avoid falls, institutionalisation, and disability (207). Furthermore, because muscle power is an important predictor of functional capacity, strategies to develop skeletal muscle power in this population must be included in any program to prevent or postpone functional limitations and subsequent disability (69, 206, 208).

### Role of physical activity and exercise in mental health

Physical activity and exercise are associated with more positive psychological attributes and a lower prevalence and incidence of depressive symptoms which are most significant in those with comorbid illnesses such as cardiovascular, pulmonary disease or major depression (209, 210, 211). Benefits have also been shown in some trials for schizophrenia (212) post-traumatic stress disorder, anxiety and other serious mental health conditions, although the data are heterogeneous and more robust trials are needed (213).

However, evidence for exercise as an isolated intervention for treating clinical depression in both younger and older cohorts is robust and consistent. Both aerobic and resistance training exercises have produced clinically meaningful improvements in depression in such patients, with response rates ranging from 25 to 88% (214, 215). In the studies addressing exercise modalities, resistance training was equivalent to aerobic training in young adults with depression, and yoga was as effective as aerobic exercise. Blumenthal et al. (211) directly compared moderate-intensity aerobic exercise with antidepressant medications in older adults with major depression and found the two approaches to be equipotent, with no added benefit of the combination of exercise and medication, and better long-term remission rates in exercise alone compared to the combined intervention or medications (211). In another study by Singh et al. (210), which compared high-intensity progressive resistance training with low-intensity progressive resistance training in individuals with major depression, a clinical response (50% reduction in Hamilton Rating Scale for depression) was achieved in 61% with high-intensity training, 29% with low-intensity training and 21% in a control group receiving usual General Practitioner care. Low-intensity PRT was not different to the control condition, with these two groups exhibiting a response rate slightly less than that reported with placebo administration in drug trials of depression (210). Similarly, low-intensity aerobic training in older adults with depression is similar in efficacy to social contact controls, reducing depression scores by only 30% (214). Depression has also been shown to require adequate doses of exercise, with lower volumes ineffective. Aerobic exercise at a dose consistent with public health recommendations (not in a group setting) was an effective treatment for major depressive disorder of mild to moderate severity, with a lower dose comparable to placebo effect (216). Thus, the literature on exercise and depression suggests that it is effective in younger and older adults, it has clear dose-response effects, is effective without group classes, and it is at least as effective as antidepressants in clinical cohorts, with lower relapse rate. Both aerobic and resistance modalities appear equally beneficial, and optimal responses are seen with higher training intensities (PRT) or volumes (aerobic).

### Effects of physical activity and exercise interventions on geriatric syndromes

#### Frailty and Sarcopenia

Frailty is a state of decreased physiological reserve that makes individuals vulnerable to stress, potentially resulting in disability and increased mortality. Its multisystem and multidimensional aspects include cognitive, social, and physical domains. Sarcopenia contributes to physical frailty. Sarcopenia is defined as a progressive age-related loss in muscle mass, strength and quality affecting physical performance. Both frailty and sarcopenia are associated with many adverse outcomes including falls, disability, cognitive decline, and mortality (29, 37, 217).

Resistance training programmes or multicomponent exercise interventions inclusive of robust resistance training have been shown to improve muscle strength in older adults with frailty and sarcopenia (69, 77, 218, 219, 220). Several intervention studies showed that exercise as a single-component or part of a multicomponent intervention can prevent/attenuate frailty (221). Most of these studies included either resistance training only or combined aerobic training with resistance and/or balance training and the intensity of exercise (both aerobic and resistance-based exercise) was low to moderate-intensity. Therefore, it is likely that they do not necessarily represent the maximum achievable adaptations, such as those reported with High-intensity resistance training (70).

Programs consisting of home-based exercise interventions, weight-bearing exercises, or very low workloads are much less effective for achieving strength gains (111, 222, 223), or treating sarcopenia and its sequelae than higher intensity prescriptions. The use of subjective scales of perceived exertion instead of strength testing to guide the progression of loads during resistance training in older adults with frailty is another factor that may result in insufficient overload of muscles and consequently reduce the magnitude of physical adaptations (224). This can be improved by ensuring that the trainer/ therapist does a simultaneous “objective” rating of exertion by evaluating the individual's breathing, Valsalva, muscle tension, or tremor, in order to override a subjective report that overestimates the true effort. The most common problem in the application of PRT is failure to adhere to DeLorme's basic founding principles, which requires continuous, progressive overload by an “uncustomary” force (225).

#### Falls

Multicomponent exercise programmes including combinations of resistance training, balance and/or gait retraining (69, 105, 111); less commonly, resistance exercise alone (74) or an alternative exercise intervention such as Tai Chi (157, 226, 227) or dance programs (228, 229, 230) have been shown to reduce falls in older adults. Aerobic training alone, by contrast, has been shown to increase falls and fracture risk (45).

Based on the evidence that such multicomponent exercise interventions are more effective in improving most, if not all, of the frailty syndrome hallmarks (i.e., poor balance, reduced muscle strength, poor gait ability, and increased incidence of falls), it is the recommended strategy for this condition. Current evidence, however, on reducing falls with physical training among community-dwelling older adults with cognitive impairment (i.e., any stage of Alzheimer's disease and related dementias, or mild cognitive impairment) is insufficient at this time to inform evidence-based recommendations or treatment decisions for clinical practice (231).

Multicomponent exercise interventions, including resistance training, gait retraining, and balance exercises, among others (e.g., occupational therapy) can be prescribed to prevent the onset of the frailty syndrome in older adults, and in people with pre-frailty (27, 69, 224, 232). Recently, the Vivifrail Project, an EU-funded project that is part of the Erasmus+ programme, is a an example of a multicomponent exercise prescription which has focused on providing training and educational materials to promote and prescribe physical exercise in older adults at risk of falls and frailty (124, 125, 233) (http://www.vivifrail.com).

In addition, because of the strong associations between functional capacity test performance, muscle power output, and rate of force development in the healthy older adult (83, 86, 208, 234), explosive resistance training (power training) has emerged as an essential intervention to improve functional capacity in older adults, including those who are frail (25, 27). Indeed, in a 12-week multicomponent exercise programme enrolling institutionalised frail nonagenarians and including a moderate-intensity power training (e.g., or at least the intention of moving fast) using a leg-press machine improved muscle cross-sectional area, muscle fat-infiltration, maximal strength and power, balance, gait, sit-to-stand ability, along with a reduction in the incidence of falls (44, 69). Therefore, explosive resistance training should always be included, if possible, in exercise interventions to improve older adults with frailty and reduced functional capacity.

One potential adverse event related to muscle power training is the potential for injury to tendons/cartilage, particularly of the rotator cuff and knee, where degenerative tears are commonplace (96), and exacerbations of abdominal/inguinal hernias (92). Interestingly, a systematic review of the effects of resistance training in frail older adults reported only one case of shoulder pain related to resistance training interventions out of 20 studies and 2544 subjects (105). Notwithstanding, to prevent injuries that could interrupt the exercise programme and its benefits, screening for such problems is critical. Care must be taken regarding the workload, volume progression, heavy and repetitive workloads, and unfavourable positioning (such as an overhead or military press or lat pulldown in rotator cuff disease). The use of moderate to high loads during power training may minimise this risk without compromising the desired outcomes of strength and power. This is because the external achieved peak velocity is lower, although it is well known (235) that if the cognitive intent to move at maximal speed remains even during isometric contractions, this optimises neural recruitment of fast twitch fibres. This submaximal external velocity when power training with moderate-high loads attenuates the ballistic nature of the movement at end range, due to the higher resistance. This is particularly important for exercises such as chest/bench press or knee extension in older adults with underlying degenerative arthritis.

#### Cognitive Impairment

A growing body of observational data and experimental evidence reveal that PA can significantly influence a wide range of cognitive functions (58, 119, 236, 237, 238, 239]). For example, in a prospective cohort study, walking has been shown to reduce the risk of dementia in a dose-dependent fashion, with a 1.8-fold increased risk for those who walked less than 0.25 miles per day as compared with > 2 miles per day, controlling for other possible risk factors (240). Age-related cognitive dysfunction might be partially mediated by suboptimal and diminishing participation in PA across the lifespan. Reduced PA can also be a precursor for cardiometabolic diseases, particularly hypertension and insulin resistance, well-known contributors to cognitive impairment.

Exercise has many beneficial effects on brain health. Exercise attenuates cognitive decline, where its effect may be partially mediated through myokines and brain-muscle crosstalk. Potential mechanisms by which exercise could improve cognitive function include increases in cerebral blood flow, increased neurotrophic factors (BDNF, insulinlike growth factor-1 [IGF-1]), downregulation of neurotoxic factors (insulin, C-reactive protein, cortisol, interleukin-6 [IL-6]), and other inflammatory cytokines, prevention or better control of chronic diseases (e.g., hypertension, stroke, diabetes, cardiovascular disease) and prevention of depression (241). High-intensity progressive resistance training has been shown to produce beneficial long-term structural brain changes after progressive resistance training in older adults with via MRI and fMRS (242, 243), including increased posterior cingulate cortex thickness, decreased white matter hyperintensity volume, and diminished atrophy of the hippocampus sustained 12 months after training cessation.

While aerobic exercise was previously recommended as the primary modality to improve cognition, recent studies have suggested potentially important roles for resistance exercise and mind-body exercises (e.g., tai-chi) (239, 244, 245), as well. Indeed, resistance training is associated with improvements in reasoning and executive function. Tai Chi has shown benefits on attention and processing speed, and Baduanjin (movement exercise) improved general memory and its sub-domains (i.e., immediate memory and delayed memory), executive function, and processing speed (237, 245, 246). In the SMART study (120), high-intensity resistance training for 6 months improved global and cognitive function in older adults with MCI compared to sham exercise (calisthenics). This benefit persisted over 18 months. The changes in lower extremity strength mediated 64% of the benefit in executive function in the training group [58], suggesting an underlying anabolic mechanism linking brain and skeletal muscle adaptations, as yet to be defined.

Exercise slows the decline or improves cognitive function of people with Alzheimer's disease (247). Exercise has also been shown to reduce the behavioural and psychological symptoms of dementia (248). Due to significant heterogeneity in older adults with dementia, as well as very heterogeneous intervention paradigms, many studies have highlighted an improvement in physical function but not cognition in these cohorts. The optimal exercise prescription for dementia prevention or treatment is not currently defined. Critically, however, low intensity, minimally supervised, minimally progressive exercise such as that published from the DAPA trial (249)., which did not conform to well-known principles of resistance training or aerobic training in terms of intensity, volume or progression, does not improve cognition. Therefore, the exercise prescriptive elements that best address the underlying contributors to dementia (sarcopenia, low aerobic capacity, hypertension, diabetes, depression, anorexia cerebrovascular pathology, inflammation, etc.) offer the best basis on which to prescribe exercise at this point in time for dementia itself. Reduction of intensity is not necessary if supervision is provided, even when dementia is present [44, 70, 210]. Evidence-based exercise modalities that holistically address falls, frailty and sarcopenia, polypharmacy, malnutrition, depression, osteoporosis, or cardiometabolic disease, should be recommended in aged care settings or cohorts of patients with established dementia.

#### Considerations regarding physical activity and exercise for frail individuals with cognitive impairment

The cognitive frailty construct was first defined some years ago and is characterised by the presence of both physical frailty and potentially reversible cognitive impairment in the absence of dementia (250). Even in very advanced dementia in residential aged care, the potentially beneficial benefits of exercise should not be discounted. For example, after long-term use of restraints in patients with coexisting frailty and dementia, an uncontrolled trial of multicomponent exercise training comprised of moderate-intensity resistance training on a leg press, combined with walking and balance exercises, cognitive exercises and occupational therapy improved gait ability, balance, and muscle strength and reduced the incidence of falls (44). Individualised multicomponent exercise training with particular emphasis on moderate-intensity power training may be a cornerstone for individuals with frailty and cognitive impairment to simultaneously improve their physical and cognitive function (25). Other exercises that benefit this group include multicomponent and dual-task exercises (26, 117, 251).

#### Role of physical activity and exercise in the prevention and treatment of disability

Physical activity and exercise are known to influence the development and expression of disability in old age (252). Physically active adults in the Established Populations for Epidemiological Studies of the Elderly (EPESE) were more likely to survive to age 80 or beyond and had approximately half the risk of dying with disability than their sedentary peers (253). Data from the Longitudinal Study of Aging showed that PA was associated with a slower progression of functional limitations and, thereby, slower progression to ADL/ instrumental activity of daily living (IADL) disability (254). There is a substantial overlap between the identifiable risk factors for disability and the consequences or correlates of habitual inactivity including advanced age, female sex, non-Caucasian ethnicity, and lower educational level and income. Psychosocial features common to both cohorts include social isolation, low self-esteem, low self-efficacy, depressive symptoms, and anxiety. Lifestyle choices more prevalent in disabled and/or inactive adults include smoking and excess alcohol consumption. Body composition changes associated with functional decline and inactivity include sarcopenia, obesity, visceral obesity, and bone loss, with associated functional consequences such as gait instability and slowness and impaired lower extremity function and mobility. Exercise capacity is typically reduced in both conditions and may include aerobic capacity, muscle strength, endurance and power, flexibility, and balance. As most studies have not assessed the full range of factors known to be associated with disability, and many were cross-sectional observations, it is not possible to say with certainty how all these complex relationships fit together, which relationships are causal, and which risk factors are independent of each other.

In addition to the associations described earlier, chronic diseases associated with inactivity, such as obesity, osteoarthritis, cardiovascular disease, stroke, osteoporosis, type 2 diabetes, hypertension, and depression, are all risk factors for disability. In some cases, data linking inactivity to disability-related diseases are available from cross-sectional, prospective cohort studies, experimental trials, and epidemiological data (255). Disability is complex and not fully explained by deficits in physical capacity such as strength and balance. Other mechanistic pathways may be operative, including sensory function, glycaemic control, psychological constructs, and other aspects of health status.

Exercise has shown to alter the trajectory of disability in older adults with frailty as evidenced from one of the most extensive reported randomised controlled trials of exercise and disability in older adults with frailty where 704 residents of nine different nursing homes were randomised into resistance, balance, and aerobic exercise; nursing rehabilitation; or a control condition. After 17 months, residents in both types of intervention homes had significantly less decline in ADL functioning than those in the control homes (256). The LIFE multicenter randomized clinical trial in 1635 older persons at risk of disability has shown a significant reduction in risk of incident mobility disability after an average of 2.6 years of follow-up in those randomized to PA compared to health education (257).

A review of studies targeting disability in disease-specific populations such as patients with depression, cardiovascular disease, stroke, chronic lung disease, and arthritis is beyond the scope of this review. Still, there is evidence that exercise is beneficial in all these conditions as a primary or adjunctive treatment. The largest body of data exists for older adults with osteoarthritis of the knee, which is one of the commonest conditions related to disability in older adults (258). Weight-bearing functional exercises, walking, and resistance training have been used in various combinations in these studies. There is no clear indication of the superiority of anyone modality over another in the reduction of pain and disability from osteoarthritis. Notably, land-based exercise is superior to stretching and aquatic exercise, despite the common perception that these less-robust exercises are more efficacious or feasible in this cohort. It is likely that the reductions in disability observed in patients with arthritis are due to the impact of exercise on a variety of factors, including muscle strength, gait and balance, body weight, pain, comorbid disease expression, self-efficacy, and depressive symptoms, among others. There is no simple link between improvements in function or pain and fitness adaptations (258).

#### Exercise to counteract iatrogenic disease

Exercise to counteract the undesirable side effects of standard medical care is gaining increasing attention in the literature. For example, exercise would include resistance training for patients receiving corticosteroid treatment to counteract the associated proximal myopathy and osteopenia not fully addressed by bisphosphonates, or neutralising the adverse effects of energy-restricted diets in obesity or protein-restricted diets in chronic renal failure (259).

An excellent target group where both resistance and aerobic exercise can play a significant role would be older men with steroid-dependent chronic lung disease, in whom harmful combination of pulmonary cachexia, malnutrition, tobacco use, steroid myopathy, and osteoporosis produce profound wasting, osteoporotic fractures, and impaired exercise tolerance. Aerobic training will improve functional status in this clinical cohort but is insufficient to address musculoskeletal wasting (260).

The major geriatric syndromes for which exercise may be beneficial as a preventive or prevention strategy are listed in Table 4, along with the postulated mechanisms of exercise benefit and the specific modality of exercise most relevant for these outcomes.

**Table 4:** Exercise and geriatric syndromes

**Geriatric syndromes**	**Considerations for the prescription**	**Recommended exercise modality**
Frailty and Sarcopenia	• Resistance and power training: 2 to 3 sessions per week, combining slower and faster (power training) muscle actions at intensities of 40 – 80% of 1RM. • Functional exercises e.g., standing from a chair with progressive increases in loading/speed • Balance and gait exercises progressing in complexity: line walking, tandem foot standing, standing on one leg, heel-toe walking.	• Resistance training • Power training • Balance exercises • Gait retraining • Multicomponent exercise
Falls/Mobility impairments	• Resistance training aimed to improve muscle strength and power. • Balance and gait exercises progressing in complexity: line walking, tandem foot standing, standing on one leg, heel-toe walking. • Dual task exercises including dual task gait and resistance exercises (serial numbers, naming animals, etc). • Adapted Tai Chi exercises progressing in complexity. • Dance interventions may improve adherence.	• Resistance training • Balance exercises • Gait retraining/dual task training • Multicomponent exercise • Dance interventions • Tai Chi exercises
Cognitive impairment	• High-intensity resistance training combined with power training aimed to improve cognitive and functional abilities. • Walking may reduce the risk of dementia. • Dual task exercises may be beneficial to cognitive function. • Use of mirror techniques rather than complex oral instructions. Use of haptic support. • Considerations of emotional aspects such as reassurance, respect, and empathy.	• Walking • Aerobic training • Resistance training • Dual-task training

### Exercise for acute hospitalised older patients

Older adults are particularly prone to hospitalisation hazards, including immobility, delirium, and functional decline, which are often associated with increased length of hospital stay, institutionalisation, and mortality (261). Exercise and early rehabilitation programmes are among the interventions through which functional decline is likely to be best prevented in hospitalised older patients (142, 262, 263, 264, 265).

Healthcare systems are still poorly adapted to the needs of older adult patients. Low levels of in-hospital mobility are directly associated with functional deterioration at discharge and, during post-discharge follow-up (266). Physical exercise plays an essential role in preventing the functional and cognitive decline associated with hospitalisation in older adults. The benefits of exercise have been clinically, biologically, and even economically confirmed (113, 267), making exercise a valuable addition to the therapeutic arsenal. Although only a few RCTs have examined the potential benefits of exercise training for acutely hospitalised older adult patients, the effects of in-hospital exercise interventions on functional outcomes are promising even in the oldest-old people with frailty (113, 264, 267, 268). Martínez-Velilla et al. (113) and Saez de Asteasu et al. (268) showed that an individualised multicomponent exercise training programme for oldest-old individuals with frailty improved their overall functional capacity and cognition during acute hospitalisation when compared with usual care. The control group received “usual” hospital care, which included physical rehabilitation when needed. For the intervention group, exercise training was programmed in twice-daily sessions (morning and evening) of 20 minutes over 5–7 consecutive days (including weekends), supervised by a qualified fitness specialist. Each session was performed in a room equipped ad hoc in an acute care of elderly (ACE) unit. The resistance exercises were tailored to the individual's functional capacity using variable resistance training machines aiming at 2 to 3 sets of 8 to 10 repetitions with a load equivalent to 30 to 60% of the 1-RM. Participants performed three exercises involving mainly lower-limb muscles (squats rising from a chair, leg press, and bilateral knee extension) and one involving the upper-body musculature. In addition, exercise training program also included exercises adapted from the Vivifrail multicomponent physical exercise programme to prevent weakness and falls (http://www.vivifrail.com) (124, 125). This contrasts with an earlier RCT showing no significant benefits of a single in-hospital mobility programme and a behavioural strategy to encourage mobility in older patients and their ability to perform ADLs after an acute hospitalisation (269). These findings suggest that interventions beyond simple walking are needed to preserve or increase functional capacity in older patients during acute hospitalisation. This is likely because muscle mass tends to decrease rapidly in older adults during hospitalisation, and the resulting loss of muscle strength and mass are associated with disability, morbidity, and mortality (270). Therefore, an individualised physical exercise intervention including low-moderate intensity progressive resistance training is an effective therapy to counteract muscle strength and mass losses that frequently occurs during hospitalisation (113, 268, 271). Once discharged, however, progression to moderate-high intensity resistance training is required to achieve the benefits shown in many of the randomised controlled trials described earlier (5, 68, 158, 210, 272, 273), given the well-described dose-response effects related to intensity and adaptations to anabolic exercise. More recently, Kitzman et al. (264) in a diverse population of older patients hospitalised for acute decompensated heart failure, an early, transitional, tailored, progressive rehabilitation intervention that included multiple physical-function domains resulted in greater improvement in physical function than usual care.

In acutely ill hospitalised patients, the exercise prescription can follow the above-mentioned exercise recommendations for older individuals with frailty. However, because these patients are at near-constant bed rest, exercise and resistance training are especially important to minimise deconditioning, and it should be performed daily if the medical condition allows until their hospital discharge. To make it more tolerable for patients, the training sessions can be split into two sessions (i.e., morning and afternoon) (124, 125). Additionally, it is crucial to pay special attention to vital signs or fluctuating mental status suggestive of delirium, infection, hemodynamic instability, or drug effects before performing the physical exercise intervention to prevent adverse events.

## Inter-individual variability and dose-response heterogeneity to physical activity and exercise

Dose-response heterogeneity is not unique to pharmaceutical therapies (274). Like the significant heterogeneity in physiological decline with age, there is also an inter-individual variability in the response to exercise. Dose-response relationships between changes in fitness and improved health outcomes have been identified for some, but certainly not all, diseases and syndromes. Concerns have been raised about the true magnitude of response variability as well as maximal trainability. In recent years, this area of investigation has become critical for defining threshold and optimal levels of activity necessary for health promotion and disease management (275). It should be recognised that what is suitable for prevention may be entirely inadequate for treatment, as is also the case with pharmacological management of chronic diseases.

As with other concepts of precision medicine, interindividual variability in the magnitude of response to supervised exercise training (subject-by-training interaction or individual response) has received increasing attention (276, 277, 278, 279, 280). For instance, some individuals show improvements with exercise training (e.g., decrease in fasting glucose) and are considered responders. In contrast, others may not have such a response (e.g., no change or even increases in fasting glucose), and are considered non-responders (138). However, these “non-responders” may adapt normally in other domains. This body of work also indicates that nonresponse to aerobic exercise is a commonly observed phenomenon in research studies with older adults (prevalence of 1.4% to 63.4%), compared with younger individuals (17% to 19%) (281, 282). The HERITAGE Study has shown that gains in ˙aerobic capacity (VO2max) demonstrate considerable interindividual variation even in response to standardized exercise training programmes in healthy adults. The estimated VO2max to account for the total trainability variance. A genome-wide association study suggests that VO2max trainability is influenced by multiple genes, each with minor effects, but much remains to be elucidated about this variability in the response (283).

Even less is understood about the prevalence of variability in the response to exercise among older adults and the factors responsible for such variability, particularly in populations of older adults with chronic conditions (284). Some modalities or doses of exercise promoted (mild calisthenics, slow-paced walking) have little or no discernible effects on physical fitness but may yield benefits in other domains. Contributing to this evidence, recent studies have demonstrated that older hospitalized patients (mean age 87 years) presented a higher inter-individual variability in the response to physical exercise than usual care during hospitalisation: functional capacity (∼15%), gait velocity (∼49%), and muscle strength (∼38%) (271). While supervised physical exercise intervention reduced the prevalence of hospital-associated disability in acutely hospitalised older adults, a high proportion of patients receiving the intervention showed no improvement (∼40%) or even an impairment (∼10%) in activities of daily living during hospitalisation (14, 271). Moreover, those adverse responders to usual-care and exercise programmes had higher one-year mortality rates after discharge (271). These results further demonstrate the considerable individual variability in the response to an in-hospital intervention for very old patients.

Many other important clinical outcomes also demonstrate heterogeneity in the response rates of older adults. For example, among community-dwelling adults, data from the HERITAGE Family Study, wherein 316 women and 280 men (173 blacks and 423 whites) healthy sedentary individuals completed a 20-week exercised on cycle ergometers 3 days per week for 60 sessions, ∼42% of participants showed no change whereas others had an adverse response in glycaemic control indices (138). Similarly, a secondary analysis of the Effects of Aerobic Exercise for Treating Alzheimer's Disease (FIT-AD) in community-dwelling older adults with mild-to-moderate dementia due to Alzheimer's disease showed true inter-individual differences in aerobic fitness and cognitive responses to aerobic exercise (285). In another medical condition, Whipple (286) reported a high prevalence of non-responders among individuals with peripheral artery disease with or without type 2 diabetes mellitus completing at least two-thirds of their prescribed exercise sessions. When non-response was defined as a negative change or no change in 6-minute walk test distance, the prevalence of non-response was 35%, but when defined as a lack of a clinically meaningful change (20 meters), the prevalence of non-response was as high as 56% (287). All participants improved in at least one study outcome, but only one individual improved in every measured outcome.

Hypothesized reasons for nonresponse include insufficient training stimulus (i.e., intensity or specificity of intervention), sex-related differences in response to exercise, and baseline fitness levels. Additionally, the individual interaction of physiological, molecular (i.e., genetics, epigenetics, transcriptomics, and metabolic factors), and environmental factors are being investigated as potential mediators of the lack of a response to exercise in some participants (288). For example, the heritability estimates for general muscle strength have been reported to range from 30% to 60%, and overall heritability of strength-related phenotypes has been estimated to be around 50% (289). Similarly, the seminal HERITAGE Family Study reported a maximal heritability estimate of 47% (and maternal heritability of 28%) of the individual cardiorespiratory fitness responses following 20 weeks of supervised and standardised aerobic exercise training (290). More than 200 polymorphisms have been associated with strength/power phenotypes, especially concerning athletic performance (291). For example, individuals with PPARGC1A gene codes for the peroxisome proliferator-activated receptor-gamma coactivator-1α (PGC-1α) Gly482Ser (rs8192678) CC genotype had lower one-repetition maximum at baseline compared to both CT genotype counterparts and T-allele carriers (292), but demonstrated that regardless of gender, baseline strength and age, 8 weeks maximal strength training intervention is effective in improving maximal strength in most healthy people. A systematic review of genetic influences on functional adaptations to aerobic or resistance training in older adults identified 7 studies measuring 10 single-nucleotide polymorphisms and 9 different functional performance test outcomes. The ACE (D) allele, ACTN3 (RR) genotype, UCP2 (GG) genotype, IL-6-174 (GG) genotype, TNF-α-308 (GG) genotype, and IL-10-1082 (GG) genotype all predicted significantly superior adaptations in at least one functional outcome in older men and women after prescribed exercise or in those with higher levels of PA (188). Much more research is needed to explain the role of these potential genetic influences on the myriad of factors related to peak functional performance. Identifying genetic predictors of blunted adaptations to exercise could potentially improve our ability to target individuals at risk for poor outcomes by recommending advanced training techniques, better behavioural strategies, or physiological augmentation via nutritional or pharmacological co-interventions.

New techniques are also needed to advance our understanding of the heterogeneity in responsiveness to exercise. For example, repeated crossover, sequential multiple assessment randomized trials (SMART), or a multiphase optimisation strategy trial (MOST) design could be used. These designs are uniquely suited to investigate the best tailoring variables and decision rules for an adaptive intervention (293). Additionally, the argument that non-response to exercise is common among older adults (and not just a result of poor repeatability or reliability in outcome measures or inadequate dosing or adherence) must be examined as a primary study outcome. This could be particularly relevant for older adults, given the increased heterogeneity among older adults with respect to health, physical function, work, leisure activities, psychological attributes, and social environment.

Despite the heterogeneity described above, one should consider that a clear dose-response relationship exists between exercise intensity and, in some cases, volume for most clinical outcomes. Therefore, there is a need to recommend evidence-based doses of exercise when treating older adults. If an older adult does not get stronger in a PRT program, it is much more likely that prescriptive principles have been violated (225) than it is that they are true “non-responders” to PRT. Thus, further investigation is warranted to evaluate whether response heterogeneity differs across population subtypes and with similar lifestyle modifications to move closer to a personalised lifestyle medicine that optimises changes in clinical outcomes based on individual characteristics (294).

## Conclusions

Insufficient PA/exercise and excess sedentary behaviours are potent risk factors for all-cause and cardiovascular mortality, obesity, sarcopenia, frailty, and disability, among other chronic diseases associated with ageing. Being physically active and having a healthy diet (coupled with no smoking and moderate alcohol consumption and the maintenance of appropriate body mass) are integral to maintaining health and well-being at all ages. Exercise and PA offer clinical benefits across a wide range of diseases and disabilities with no upper age limit (13). An appropriate exercise prescription should be included in all healthcare recommendations in the effort to enhance functional independence, psychological well-being, and quality of life through the promotion of exercise for all older adults, whether fit or frail, of any age (13, 32). Physical activity (including structured exercise participation) influences key drivers of ageing even in the oldest-old, including chronic inflammation, mitochondrial dysfunction, myokine release, autophagy, oxidative damage, and insulin-like growth factor signalling. Exercise and PA improve physical function and quality of life, reduce the burden of non-communicable chronic diseases and premature overall mortality including cause-specific mortality from cardiovascular disease, cancer, and chronic lower respiratory tract diseases. The beneficial effects of exercise are global (i.e., acting at both the physiological multisystem and functional capacity level). Exercise interventions are currently more useful than pharmacological interventions that target single systems (e.g., inflammation or anabolic hormones) for managing frailty. Declines in muscle function and cardiorespiratory fitness with ageing result in impaired ability to perform daily activities and maintain Independence. Accordingly, the treatment of frailty should be focused on improvement in overall functionality, complementary to the diagnosis and treatment of specific diseases.

Exercise is medicine, and the prescription of PA/structured exercise should therefore be based on the intended outcome (e.g., primary prevention, improvement in fitness or functional status or disease treatment), and should be individualised, and controlled like any other medical treatment. The prescription of exercise for health-related outcomes must consider not only dose-response relationships with volume and intensity, but also modality-specific adaptations which are requisite for certain outcomes to be achieved. Since most patients will present with more than one disease, an efficient prescription to optimise both safety and efficacy is mandatory as described in this consensus statement. Importantly, beyond its practical benefits, long-term physical exercise results in less harm among older adults than maintaining usual activity levels or usual care, independent of participants' age, physical function, or cognition status at baseline. Considering the accumulated evidence of the benefits of exercise in frail older adults over many decades, it is not justifiable to not prescribe physical exercise to these individuals, and one of the main challenges for the future is to integrate exercise programmes as a mandatory part of the care of pre-frail/frail older patients in all hospital, outpatient, and aged care settings (32).
